# A Cable-Driven Hybrid Robot with Series-Parallel Coupling: Design, Modeling, Optimization Analysis, and Trajectory Tracking

**DOI:** 10.3390/s26041147

**Published:** 2026-02-10

**Authors:** Zhifu Xue, Zhiquan Yang, Junyi Hu, Bin Zhu, Jianqing Peng

**Affiliations:** 1 School of Intelligent Systems Engineering, Shenzhen Campus of Sun Yat-sen University, Shenzhen 518107, China; xuezhf@mail2.sysu.edu.cn (Z.X.); yangzhq33@mail2.sysu.edu.cn (Z.Y.); hujy68@mail2.sysu.edu.cn (J.H.); zhub26@mail.sysu.edu.cn (B.Z.); 2 Guangdong Provincial Key Laboratory of Fire Science and Technology, Guangzhou 510006, China

**Keywords:** cable-driven hybrid robot, force-closure workspace, series-parallel coupling, self-calibration, trajectory planning

## Abstract

Compared to purely serial robots or cable-driven parallel robots (CDPRs), cable-driven hybrid robots (CDHRs) combine the advantages of both, addressing their limitations and enabling the execution of complex tasks. The series-parallel coupling structure increases the complexity of the system, complicating modeling, calibration, and force-closure workspace (FCW) analysis. This study develops a CDHR system equipped with various sensors and proposes methods for series-parallel coupling modeling, workspace analysis, and self-calibration of complex systems. First, the modular design requirements for the CDHR are analyzed, comprising an 8-cable parallel drive and a 4-degree-of-freedom serial manipulator. Second, a kinematic model of the CDHR with series-parallel coupling was derived, and the positions of the dynamic anchor seats were optimized using an optimization algorithm. Based on these optimized results, a modeling and analysis method for the statics and FCW is proposed. Furthermore, considering the complex and interdependent structural parameters of the system, a method for the self-calibration of the system parameters and trajectory planning for the CDHR is presented. Finally, experimental validation on both simulations and a physical prototype confirmed the effectiveness of the proposed methods. The developed prototype and the proposed method provide a basis for high-precision operations in large spaces, operations in dangerous/extreme environments, and automated operations in logistics/warehousing.

## 1. Introduction

The cable-driven parallel robot (CDPR) replaces traditional rigid links with a cable traction mechanism, reduces structural inertia, and achieves a higher payload-to-weight ratio, increased speed, and an expanded workspace [1,2,3]. By incorporating a serial manipulator (SM) at the end, the degrees of freedom (DOFs) were further increased, allowing adaptation to diverse manipulation tasks. Thus, a cable-driven hybrid robot (CDHR) combines a CDPR with a serial manipulator (SM) [4]. This hybrid design overcomes the limitations of each individual robot and enables more complex tasks, offering a high payload-to-weight ratio, a large workspace, and high operational efficiency. Consequently, CDHRs have broad application prospects in unstructured and complex environments such as disaster rescue, lifting operations, and large-scale equipment maintenance [5].

**Figure 1:**
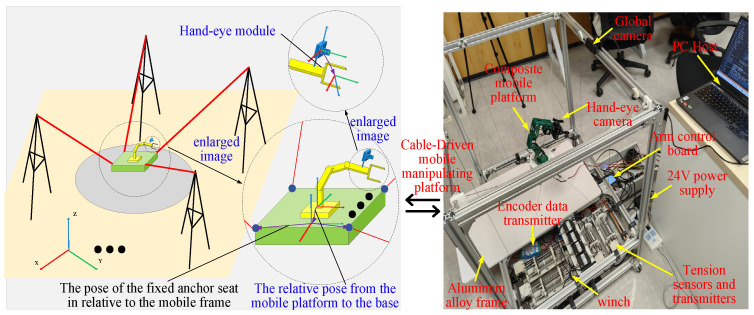
The overall structure of the cable-driven parallel robot.

Significant progress has been made in the design and modeling of hybrid robots. As shown in Figure 1, a hybrid robot was formed by mounting an SM on the mobile platform of the CDPR [6,7]. This architecture combines the CDPR’s large task-space workspace with the SM’s high local end-effector dexterity [8,9,10]. As a result, it improves both global reach and fine motion capability near a given pose. However, this also makes its modeling and control more challenging. The kinematics of the CDHR can be decomposed into those of the CDPR and SM. Because the generalized joint DOFs are significantly higher than the 6-DOFs at the end effector, the kinematic solutions of the CDHR exhibit a high degree of redundancy.

Pinto et al. [11] designed a CDHR that attaches a rotary gripper as an end-effector tool beneath a mobile platform, thereby enhancing both the spatial range and flexibility of the object manipulation. Osumi et al. [1] proposed a planar CDHR structure that is suspended from a slider and achieves single-DOF reciprocating motion via a linear guide rail. A cable-driven platform was mounted below the slider and carried a 4-DOF SM. For this planar CDHR, an equivalent centroid modeling method was proposed [12]. In that model, cable torques and manipulator moments were neglected. El-Ghazaly et al. [13] designed a hybrid cable-thruster (HCT)-actuated underwater vehicle-manipulator system (UVMS) comprising an underwater operating platform and manipulator, and developed a dynamic modeling method that considers buoyancy and platform weight. Gao et al. [14] analyzed the kinematics of a spring-supported CDPR, integrating statics with inverse kinematics (IKs) to achieve rapid solutions. Lau et al. [15] released CASPR, an open-source software that incorporates various kinematic modeling methods for CDPRs.

To investigate the stability and performance metrics of CDPRs throughout the workspace, scholars have recently proposed methods based on Monte Carlo random sampling, convex hull theory, and other techniques to study force-closure workspaces (FCWs) [16,17]. The FCW of a CDPR is defined as the set of pose points within the kinematically reachable space, where the moving platform can remain stationary under cable tension [18]. In a six-dimensional Cartesian space, the generalized forces exerted by the cables include both forces and torques. Hence, the FCW is also referred to as the wrench-closure workspace (WCW). The FCW is a crucial performance metric for controlling CDHRs [19,20].

Pham et al. [15] proposed an FCW solution method based on the dimensionality reduction in the cable structure matrix using Gaussian elimination. This approach projects high-dimensional vector sets onto lower-dimensional ones and determines whether the moving platform can satisfy static equilibrium conditions by checking the force closure of all lower-dimensional vector sets in the projected space. Diao et al. [17] established the coupling relationship between the structure matrix and cable tension for a 7-DOF CDPR and calculated the FCW by evaluating the existence of solutions at various pose points. However, this method is only applicable to fully constrained 6-DOF CDPRs. Arai et al. [21] studied the workspace of a two-dimensional suspended hybrid manipulator, where the cable-driven platform provides tension to the base of the SM. The experimental results indicated that when the cable lengths were held fixed (i.e., the platform was not actuated), the end-effector’s wrench-feasible workspace was reduced compared with the case of active cable adjustment. Yang et al. [18], using a high-dimensional vector decomposition approach, transformed the force-closure determination problem into a two-dimensional vector enclosure problem around the origin and voxelized the sampling space to solve for the FCW volume. They further introduced the Tension Factor (TF) and Global Tension Index (GTI) as indicators for evaluating workspace quality, which can serve as performance metrics for robot design. Osumi et al. [22] developed a planar CDHR system consisting of a suspended 4-DOF manipulator, where joint angles were constrained within certain ranges due to the hybrid mechanical structure, leading to partial workspace loss. From a review of FCWs for CDHRs, it is evident that there are currently few analysis methods that address workspaces with upper and lower bounds of cable tension or mechanical collision constraints [23]. Most previous studies have focused on planar CDHRs. Therefore, research on FCWs of CDHRs under multiple constraints and with higher DOFs is crucial.

After assembling the CDPR, system calibration is required for trajectory planning and control of the CDHR. Owing to the unknown initial cable lengths, the calibration model must incorporate unknown initial joint angles. The forward kinematics (FKs) of the CDHR cannot directly provide explicit results, nor can they be directly converted into a homogeneous transformation matrix (HTM). Therefore, the positions of the anchor seats must first be identified to obtain the HTM between moving and global frames.

Scholars have proposed traditional external instrument calibration and self-calibration methods to identify the position parameters of anchor seats in CDPRs [24]. Traditional calibration methods use high-precision measurement devices, such as inclinometers or laser rangefinders, to measure observable quantities in kinematic equations. The errors were then mapped to the kinematic parameters to be identified using the Jacobian matrix, enabling parameter correction. Self-calibration uses a robot’s internal sensors to correct kinematic parameters. Borgstrom et al. [25] defined the anchor seat model as a pinhole model and formulated the identification model as a mathematical optimization problem, where the objective is to minimize the squared difference between the fitted and actual cable lengths based on the estimated coordinates of the anchor seats. However, this method does not consider the physical model of the cable-anchor seat connection. Zhang et al. [26] incorporated the effects of rotating pulleys at the anchor seats in their kinematic modeling of CDPRs and established a mapping between cable length errors and anchor seat position errors. They generated an identification matrix and continuously corrected the position errors of anchor seats using an iterative optimization method. Yuan et al. [25] developed a mapping relationship between motor angle errors and anchor seat errors, using an iterative optimization method to correct the position errors of the anchor seats. Wang et al. [27] abstracted kinematic parameter identification into a general calibration model and analyzed the impact of errors on the coefficient matrix of nonlinear equations based on matrix perturbation theory. They also introduced initial joint angles as unknown parameters in the calibration equations and experimentally validated their method on a 6-DOF CDPR. Ma et al. [28] proposed a probabilistic method for solving the “***AXB = YCZ***” equation. Peng et al. [29] developed a hybrid calibration model for cable-driven hyper-redundant robots and solved it using a particle swarm optimization (PSO) method. If kinematic parameter calibration is conducted separately for each structure of the CDHR, each reconfiguration requires step-by-step calibration, resulting in high time costs. To achieve fast and accurate calibration, the challenge of simultaneously calibrating of multiple unknown mappings must be addressed.

As shown in Table 1, we summarize and compare different modeling, workspace analysis, and control methods. Although CDHRs have made some progress, several challenges remain. First, the strong series-parallel coupling of CDHRs complicates the resolution of their dynamics. Second, kinematic parameter identification for reconfigurable CDHR systems is complex and inefficient. Finally, there is complex force coupling between the CDPR and SM, where changes in the motion state of one affect the other nonlinearly, resulting in poor control accuracy and stability. To address these issues, this study designs a CDHR and derives a generalized pose kinematic and static model that considers the series-parallel coupling relationship. Furthermore, the FCW of the CDHR was solved based on this model and a study on simultaneous calibration and trajectory tracking control considering multiple mapping relationships was conducted.

The main contributions of this study are as follows:

(1) A modular, highly integrated, and compact prototype of a CDHR was developed that is capable of rapid self-reconfiguration and cable tension control. Unlike traditional designs, this system utilizes a distributed control architecture (STM32 and Arduino) with integrated tension and angle sensors, enabling effective cable tension management and creating a physical platform for validating complex coupling operations.

(2) A kinematic and static modeling method for the series-parallel hybrid structure of the CDHR was proposed. Unlike traditional approaches that often simplify inter-component dynamics, our model meticulously accounts for the strong nonlinear coupling effects between the cable tensions, the platform pose, and the serial manipulator’s joint configurations. This involved deriving explicit expressions for both the forward and inverse kinematics that consider the variable anchor points and the cable length constraints through iterative methods, coupled with a static model that accurately predicts the force distribution within the redundant cabling system.

(3) A workspace analysis method and dynamic anchor seat position optimization for the CDHR were introduced. We proposed a coupled workspace analysis method utilizing Gaussian boundary enrichment to precisely determine the Force-Closure Workspace (FCW) under complex series-parallel constraints. Furthermore, a dynamic anchor seat optimization strategy based on the global condition number was implemented using the Particle Swarm Optimization (PSO) algorithm, significantly enhancing the robot’s kinematic isotropy and minimizing collision risks.

(4) A self-calibration method for the structural parameters and a trajectory tracking method for the CDHR were presented. We developed a vision-based self-calibration method that simultaneously identifies the fixed anchor seat coordinates, the relative pose of the serial manipulator, and hand-eye parameters. Experimental validation demonstrated that this method reduces the maximum position error to 0.10 mm and the orientation error to 0.07°, significantly improving the initialization efficiency and static accuracy of the system compared to step-by-step calibration approaches.

The remainder of this paper is organized as follows. Section 2 introduces the mechanical design and system setup of the prototype. Section 3 derives the mathematical model of the series-parallel coupling structure. Section 4 and Section 5 analyzes the workspace of the CDHR and optimizes the position of dynamic anchor seats using an optimization algorithm. Section 6 and Section 7 focuses on the self-calibration of the structural parameters of the system and conducts trajectory-tracking control based on calibration results. Section 8 presents the simulations and experiments to validate the methods and prototypes described in this paper. The final section provides conclusions and outlines future work.

## 2. Design of the Hybrid Robot

### 2.1. Function Analysis and Prototype Design

To satisfy the application requirements of large-space movement and flexible operation in local spaces for complex scenarios, a CDHR prototype with an 8-cable parallel drive and a 4-DOF serial joint linkage was designed. To enhance the global flexibility of the moving platform within the workspace and the dynamic collision constraints between the cables and the robot itself, the distribution of the cable connection anchor points on the moving platform was optimized, and a new type of moving platform with an SM mounted at the end effector was designed. The hybrid robot is structured into two spatial layers: the upper and lower layers are used for motion demonstration and hardware placement, respectively, eliminating the need for an additional drive box and improving the space utilization. Each winch module is equipped with a tension sensor and an angle sensor to achieve cable pretension control, overload alarms, and cable position feedback.

**Figure 2:**
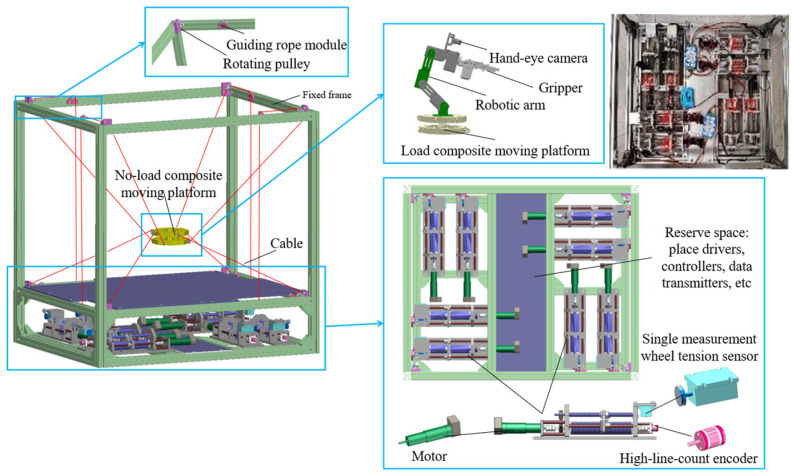
**Three-dimensional** structure of the prototype.

This study analyzed the functionality of the prototype across three modules: hardware design, algorithm design, and system design. In the hardware design module, the need for large-range spatial movement of the moving platform is addressed by designing an 8-cable-driven hybrid platform. Simultaneously, to perform complex operations in local spaces, a flexible manipulator was mounted on a moving platform with a vision sensor installed at its end. In the algorithm design module, the coupling forces between the moving platform and manipulator base must be calculated, and a coupled kinematic model is established. In addition, a cable tension control algorithm was designed to maintain the stability of the platform and cables. In the system design module, the lower-level controller reads the sensor data, controls the motors, and communicates with the upper-level controller, which sets up the simulation environment, develops a visual user interface, and communicates with the lower-level controller. Based on these considerations, the designed CDHR prototype is illustrated in Figure 2. The prototype has overall dimensions of 860 mm × 705 mm × 850 mm and consists of a frame, hybrid moving platform, motors (i.e., MAXON RE 25 [M10.1], **maxon motor ag, Sachseln, Switzerland**), drivers (i.e., Robomodule, **Shenzhen RoboModule Technology Co., Ltd., Shenzhen, China**), tension sensors (i.e., JZHL-M1, **Hefei Jinnuo Company, Hefei, China**), angle sensors (i.e., PD-1503-ENC-1024, **Dongguan Pudi Electronic Technology Co., Ltd., Dongguan, China**), transmitters (i.e., IBF166-WIFI-N, **Shenzhen Beifu Technology Co., Ltd., Shenzhen, China**), and winches. The prototype was equipped with eight driving cables and four serial joints to achieve 6-DOF motion by employing a distributed control method to drive the robot. The motors drive cable movements are located at the base of the prototype, whereas the motors that drive the manipulator on the moving platform are housed within the manipulator itself, thereby reducing the load on the moving platform through a separate arrangement. The hybrid moving platform is driven by cables, whereas the motors on the platform control the movement of the manipulator, enabling tasks such as recognition and grasping.

The moving platform in this prototype was driven using eight cables, with each wound around a winch located on the base. Cables were drawn from the winches and connected to the hybrid moving platform through a cable guide module and rotating pulleys. During the movement of the hybrid platform, the motors on the base drove the rotation of the winch drums, allowing the cables to be retracted or released. The angle sensors installed on the base provide feedback regarding the rotation angles of the drums, which can be used to determine the length of the cable that is retracted or released. Tension sensors provide feedback on the forces exerted on the cables. When the current cable lengths are known, the pose of the moving platform can be calculated using a fixed coordinate system. Conversely, when the desired pose of the moving platform in a fixed coordinate system is known, the corresponding cable length required to reach this pose can be determined. The effects of cable mass, inertia, and damping on solution accuracy are minimal; thus, it is assumed that the cables are subjected only to axial tension. Therefore, the movement of the hybrid platform can be controlled using feedback from cable lengths and tension forces.

### 2.2. Hardware and Software System Design

**Figure 3:**
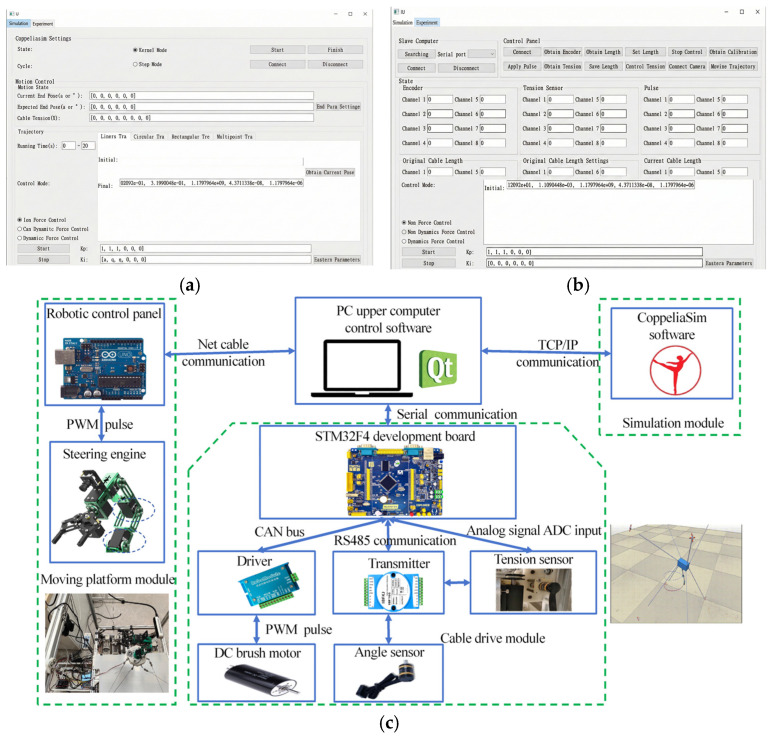
Framework of hardware and software design for hybrid cable-driven robot systems. (**a**) Trajectory tracking control interface of the upper computer, (**b**) communication interface control between the upper and lower computers, and (**c**) interaction interface between the upper and lower computers.

The entire hybrid robot system consists of an upper-and lower-level controller with sensor data acquisition based on the high-speed RS485 serial bus standard, ensuring that the data feedback cycle is significantly shorter than the control cycle. The software and hardware design frameworks of the system are shown in Figure 3. The upper-level software, including the simulation and prototype controls, was built using the PyQt5 environment with the control interface illustrated in Figure 3a. The simulation control software communicates with CoppeliaSim to monitor robot states and control movements, whereas the prototype control software communicates with the lower-level controller board to read, display, and detect sensor data. It controls the cable-driven motors of the parallel platform through serial communication from the upper-level controller and the drive motors of the serial manipulator through Ethernet communication, thereby achieving comprehensive control of the prototype. On the hardware side, the cable drives were controlled using an STM32F4 development board, whereas the movement of the manipulator was controlled using an Arduino development board. To control information feedback, tension sensors use transmitters to limit the output voltage signals to a range of 0~3.3 V. These signals were inputted to the ADC channels of STM32F4, where the voltage values were converted into cable tension through a mapping relationship. Angle sensors perform quadrature decoding of their output signals via transmitters, with data transmission between the transmitters and STM32F4 utilizing the RS485 communication protocol.

To analyze the impact of kinematic parameters on the workspace of the CDHR, a modeling and analysis method for kinematics, statics, and workspace was proposed, considering a series-parallel coupling structure. At the kinematic level, the relative position between the base of the SM and moving platform was considered. At the static level, a mechanical model accounting for the force coupling between the moving platform and base was established. Based on this static analysis, a comprehensive examination of the FCW of the CDHR was conducted. To achieve precise movement and autonomous grasping operations, a self-calibration and grasping control strategy based on visual positioning was proposed. After assembling the robot, a vision-based self-calibration method was developed to reduce the complexity of the calibration process during operation. This method simultaneously calibrates the anchor seat positions and the relative position between the moving platform and the base using visual detection data and sensor feedback from the robot. Based on the calibration results, upper-level software was developed to simulate grasping control for typical tasks. The proposed self-calibration and grasping control methods were validated using simulation software, and the experimental results demonstrated their effectiveness and accuracy. Finally, the performances of the proposed methods and prototype were validated through calibration experiments conducted on the CDHR prototype. The experiments included sensor parameter calibration, motor reduction ratio calibration, and system self-calibration, which enhanced the motion accuracy of the prototype. Additionally, cable tension control and complex trajectory tracking experiments were conducted to verify the effectiveness of the control strategies and motion performance of the robot.

## 3. Coupled Kinematic and Static Modeling

**Figure 4:**
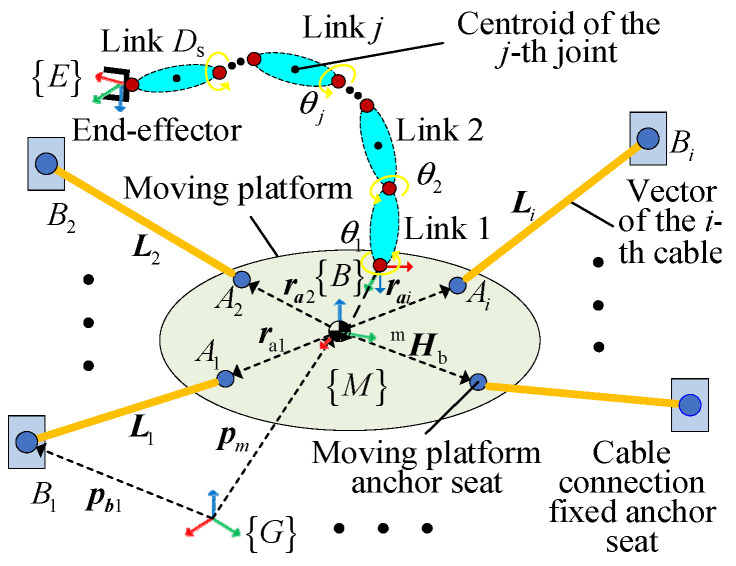
Structure of the CDHR.

The model parameters of the CDHR are defined in Table 2, and those of the CDHR with $\left(D_{c}+D_{s}\right)$-DOFs are shown in Figure 4. It is assumed that all winches of the CDHR are equal.

### 3.1. Motor-to-Cable FKs

The relationship between the motor rotation angle and the driver pulse value is:

(1) $$\Delta \theta_{w}=\frac{p_{d}}{4l_{m}r_{m}}\cdot 2\pi$$

where $p_{d}$ is the number of driver pulses, $l_{m}$ is the number of encoder lines, and $r_{m}$ is the motor gearbox-reduction ratio.

Because of the synchronous rotation of the motor output shaft and drum through coupling, the lead of the helical line on the drum, height along the axis of the cylinder, and circumference of the cross-section form a right triangle. Consequently, the relationship between the change in cable length Δl and motor rotation angle can be expressed as:

(2) $$\Delta l=\rho_{w}\cdot \Delta \theta_{w}$$

where $\rho_{w}=\frac{\sqrt{{\left(\pi d_{w}\right)}^{2}+p_{w}^{2}}}{2\pi }$ is the cable length for one winch rotation, $d_{w}$ is the cross-sectional diameter of the cylindrical drum, $p_{w}$ is the pitch of the helical groove on the cylindrical drum, $\Delta \theta_{w}$ is the motor rotation angle.

We assume that ${\bar{l}}_{i}$ is the initial length of the *i*th cable, ${\bar{\theta }}_{wi}$ is the corresponding motor rotation angle, and $\theta_{wi}$ is the current motor rotation angle. The cable length between the fixed anchor point at the directional pulley and anchor seat on the mobile platform is determined by:

(3) $$l_{i}={\bar{l}}_{i}+\left(\theta_{wi}-{\bar{\theta }}_{wi}\right)\cdot \rho_{w}$$

Equation (3) can be rewritten in vector form as follows:

(4) $$l=\bar{l}+\rho \cdot \left(\Theta_{w}-{\bar{\Theta }}_{w}\right)$$

where $\bar{l}={\left[{\bar{l}}_{1},{\bar{l}}_{2},\ldots ,{\bar{l}}_{D_{c}}\right]}^{T}$ is the vector of the initial lengths of all cables, and ${\bar{\Theta }}_{w}={\left[{\bar{\theta }}_{w1},{\bar{\theta }}_{w2},\ldots ,{\bar{\theta }}_{wD_{c}}\right]}^{T}$ represents the initial values of $\Theta_{w}$.

### 3.2. Cable-to-End FKs

Owing to the presence of force coupling, changes in the joint angles of the SM alter the interaction forces between its links, leading to changes in the coupling forces between the manipulator and moving platform system. This in turn can cause the moving platform to experience vibrations or oscillations, resulting in changes in the robot’s end-effector pose and affecting the robot’s motion accuracy. The generalized joints and generalized pose of the CDHR can be defined as:

(5) $$Q_{\mathrm{gj}}={\left[\underset{l}{\underset{︸}{\overset{\mathrm{cable}\;\mathrm{length}}{\overset{︷}{l_{1},l_{2},\ldots ,l_{i},\ldots ,l_{D_{c}}}}}},\underset{\Theta_{s}\;}{\underset{︸}{\overset{\mathrm{joint}\;\mathrm{angles}}{\overset{︷}{\theta_{1},\theta_{2},\ldots ,\theta_{j},\ldots ,\theta_{D_{s}}}}}}\right]}^{T}\in {\mathbb{R}}^{D_{c}+D_{s}}$$

(6) $$Q_{\mathrm{gp}}={\left[\underset{X_{m}}{\underset{︸}{\overset{\mathrm{platform}\;\mathrm{pose}}{\overset{︷}{x_{m},y_{m},z_{m},\alpha_{m},\beta_{m},\gamma_{m}}}}},\underset{\Theta_{s}\;}{\underset{︸}{\overset{\mathrm{jo}\mathrm{int}\;\mathrm{angles}}{\overset{︷}{\theta_{1},\theta_{2},\ldots ,\theta_{j},\ldots ,\theta_{D_{s}}}}}}\right]}^{T}\in {\mathbb{R}}^{6+D_{s}}$$

Based on the geometric relationship between the pulley and cable, the cable length is divided into two parts: a straight segment and an arc segment. The straight segment is the line between the tangent point at which the cable leaves the rotating pulley and anchor seat on the moving platform. The arc segment is a circular arc in which the cable wraps around the rotating pulley. The lengths of the straight and arc segments can be expressed as:

(7) $$\left(l_{i,s},l_{i,a}\right)=f_{\mathrm{cdpr}\_\mathrm{ik}}\left(p_{bi},r_{ai},R_{r},n_{i},X_{m}\right)$$

where $l_{i,s}$ is the length of the straight segment, $l_{i,a}$ is the length of the arc segment, $l_{i}=l_{i,s}+l_{i,a}$, $R_{r}$ is the radius of the rotating pulley, and $n_{i}$ is the axis of rotation of the pulley.

**Figure 5:**
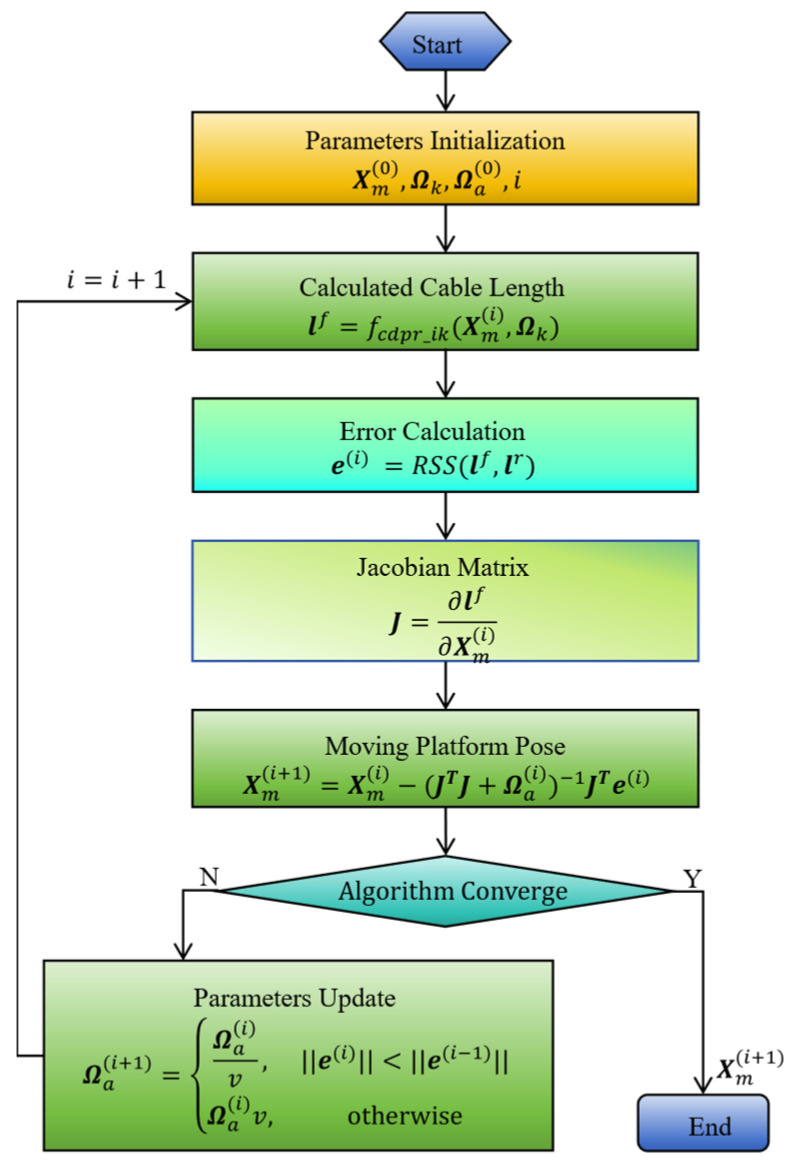
The pose resolving process of the moving platform based on the Levenberg–Marquart (LM) method. RSS(·,·) denotes the residual sum of squares. $X_{m}^{\left(i\right)}$ demotes the pose of the moving platform at step i. $\Omega_{a}^{\left(i\right)}$ demotes the algorithm parameters at step i. $e^{\left(i\right)}$ denotes the error at step i. $l^{r}$ denotes the actual cable length. $\Omega_{k}$ demotes the kinematic parameters. v denotes the adjustment factor.

According to Equation (7), the optimization model for the cable length can be established as:

(8) $$\begin{array}{l} X_{m}=\underset{\Theta_{s}\in \Phi }{\arg\min}\left(l^{r}-l^{f}\right)\\ \;\;=\underset{\Theta_{s}\in \Phi }{\arg\min}\left(l^{r}-f_{\mathrm{cdpr}\_\mathrm{fk}}\left(p_{b},r_{a},R_{r},n,X_{m}^{f}\right)\right) \end{array}$$

where $l^{r}$ represents the actual length of the cable, $l^{r}={\left[l_{1}^{r},l_{2}^{r},\ldots ,l_{D_{c}}^{r}\right]}^{T}$, $l^{f}$ is the iterative solution value of the cable length, and $l^{r}-l^{f}$ is the residual error. $X_{m}^{f}$ is the iterative solution value of the pose of the moving platform. Φ is the admissible set of the serial arm joint vector. As shown in Figure 5, using the Jacobian matrix derived from the cable lengths with respect to the pose of the moving platform, the cable length error is mapped to the pose error of the moving platform, thereby determining the actual pose.

Furthermore, the HTM of the CDHR end-effector frame relative to the SM base frame is:

(9) Teb=TDs0=T10⋅T21…TDsDs−1

where Tk+1k is the HTM from the *i*th joint to the (*i* + 1)-th joint.

Based on Equation (9), we obtain:

(10) Teg=Tmg⋅Tbm⋅Teb

where Tbm is the HTM from the moving platform frame to the base frame of the SM, and Tmg is the HTM from the global frame to the moving platform frame.

Therefore, the IKs equation of the SM can be expressed as:

(11) $${\dot{\Theta }}_{s}=J_{s}^{+}\left(\Theta_{s}\right)\cdot {\dot{X}}_{e}$$

where $J_{s}^{+}\left(\Theta_{s}\right)$ represents the Moore–Penrose pseudoinverse of $J_{s}\left(\Theta_{s}\right)$, $J_{s}\left(\Theta_{s}\right)=\left[J_{1}\left(\theta_{1}\right),J_{2}\left(\theta_{2}\right),\ldots ,J_{D_{s}}\left(\theta_{D_{s}}\right)\right]$, $J_{s}\left(\Theta_{s}\right)$ is the Jacobian matrix of the SM mapping from the joint space to the operational space, and ${\dot{X}}_{e}$ is the derivative of the end-effector pose in the global frame.

By combining Equations (8), (9) and (11), the pose $X_{m}$ of the moving platform and $Q_{\mathrm{gp}}={\left[\begin{array}{cc} X_{m}^{T} & \Theta_{s}^{T} \end{array}\right]}^{T}$ can be obtained. Therefore, the FKs equation of the CDHR can be expressed as:

(12) Xe=CFKpbi,rai,Tbm,Qgj

where $\mathrm{CFK}\left(\cdot \right)$ represents the coupled model of the FKs of the CDHR.

### 3.3. IKs of the CDHR

Similarly, defining the coordinates of the SM base relative to the global frame as $X_{b}={\left[x_{b},y_{b},z_{b},\alpha_{b},\beta_{b},\gamma_{b}\right]}^{T}$, and by combining Equations (9) and (10), the HTM of the base relative to the global frame can be written as:

(13) Tbg=Teg⋅TDs−10

Because the SM is rigidly connected to the moving platform, after the design, manufacturing, and assembly of the moving platform and the attached manipulator, the relative pose relationship Tmb remains constant after calibration. Therefore, the HTM of the moving platform relative to the global frame can be expressed as:

(14) Tmg=Tbg⋅Tmb=Tbg⋅Tb−1m

As observed from Equation (14), the pose of the moving platform depends solely on the base pose, which is determined by the joint angle. Once Tmg is obtained, the relative pose $X_{m}$ can be further derived.

Based on the above analysis, the pose estimation of the base and moving platforms can be formulated as an optimization problem. In this study, the objective function of the optimization equation was defined as the weighted sum of the deviations between the actual and desired joint angles of the arm. The deviations between the actual and desired rotational poses of the moving platform can be expressed as:

(15) Xm,Xb,Tmg,Tbg,Θs=argminXb∈X,Θs∈Φλ1∑jDsθjdes−θj+λ2αmdes−αm+βmdes−βm

where $\alpha_{m}^{\mathrm{des}}$ and $\beta_{m}^{\mathrm{des}}$ are the desired rotational angles of the moving platform along the x-axis and y-axis, respectively, $\theta_{j}^{\mathrm{des}}$ represents the desired joint angle of the *j*th joint of the manipulator, and $\lambda_{1}$, $\lambda_{2}$ are the weighting coefficients.

Furthermore, based on Equation (7), the cable length l can be determined, and the coupled model of IKs for the CDHR can be described as

(16) Qgj,Qgp=CIKpbi,rai,Θs,Tmb,Nsample

where $\mathrm{CIK}\left(\cdot \right)$ represents the coupled IKs equation.

### 3.4. Static Modeling

According to the principles of mechanics, the static equilibrium equation of a moving platform can be expressed as:

(17) $$\left\{\begin{array}{l} \sum_{i=1}^{D_{c}}T_{ci}+G_{p}+F_{\mathrm{em}}=0\\ \sum_{i=1}^{D_{c}}M_{ci}+M_{\mathrm{em}}=0 \end{array}\right.\Rightarrow \left\{\begin{array}{l} \sum_{i=1}^{D_{c}}t_{ci}{\hat{L}}_{i}+G_{p}+F_{\mathrm{em}}=0\\ \sum_{i=1}^{D_{c}}r_{ai}\times \left(t_{ci}{\hat{L}}_{i}\right)+M_{\mathrm{em}}=0 \end{array}\right.$$

where $G_{p}$ is the gravitational force acting on the moving platform, $F_{\mathrm{em}}$ is the external force applied to the moving platform, $M_{i}$ is the moment exerted on the moving platform by the tension in the *i*th cable, and $M_{\mathrm{em}}$ is the external moment acting on the moving platform.

However, because there is no relative motion between the base of the SM and the moving platform, the force exerted by the link one on the base is equivalent to the force exerted by link one on the moving platform. In the moving coordinate system, the force-coupling relationship between the base and moving platform can be expressed as:

(18) Fem=Fs=−Rbmf10Mem=Ms=Rbmτ10−tbm×(Rbmf10)Wem=Ws=FemTMemT=FsTMsT

where $F_{s}$ and $M_{s}$ represent the force and moment exerted by SM on the moving platform in frame $\left\{B\right\}$, respectively. $W_{s}$ denotes the generalized force applied by SM on the moving platform in frame $\left\{B\right\}$. Rbm and tbm represent the rotation matrix and the position vector of $\left\{B\right\}$ relative to $\left\{M\right\}$, respectively.

Substituting Equation (17) into Equation (18), we obtain:

(19) ∑i=1DctciL^i+Gp−Rbmf10=0∑i=1Dcrai×tciL^i+Rbmτ10−tbm×f10=0

Equation (19) represents the static equilibrium condition of the CDHR. Therefore, the optimization model can be established with the objective of minimizing cable tension, thus:

(20) $$\begin{array}{l} f=\frac{1}{2}\min\sum_{i=1}^{D_{c}}{t_{ci}}^{2}\\ s.t.\;A_{p}t_{c}+G_{p}+W_{\mathrm{em}}=O_{3\times 1}\\ \;\;\;\;\;\;\;\;0\leq t_{\min}\leq t_{ci}\leq t_{\max},i=1,2,\ldots ,D_{c} \end{array}$$

where $A_{p}={\left(\frac{dl}{dX_{m}}\right)}^{T}$ is the structural matrix of the cables, $t_{\min}$ and $t_{\max}$ represent the lower and upper bounds of the cable tension, respectively.

Equation (20) is a quadratic objective function with the constraints of linear equality and inequality. This problem can be solved using quadratic programming. If a numerical solution for cable tension exists, it indicates that the moving platform satisfies the static equilibrium condition and can provide sufficient support for the SM. If no numerical solution for cable tension is found, the current state cannot maintain a static equilibrium.

## 4. Coupled Workspace Modeling and Analysis

In general, the FCW of a CDPR is the set of all end-effector pose points that satisfies the static equilibrium condition, reflecting the stability of the robot within its kinematically reachable space. The force closure of a pose point can be determined by the existence of solutions to the static equilibrium equation or by the multidimensional convex hull formed by cable tension [16,19]. The convex hull formed by cable tension indicates the extent to which the moving platform can resist generalized forces in different dimensions. The force closure can be determined by checking the enclosure of each lower-dimensional projection of the convex hull; however, this method is unsuitable for CDHRs. To determine the boundary of the FCW accurately, this study used the Gaussian boundary enrichment method to densify the boundary points of the workspace [33]. Additionally, in CDHRs, the SM may collide with the overall system or the environment, making it necessary to constrain the joint angles of the SM, namely,

(21) $$\theta_{j,\min}\leq \theta_{j}\leq \theta_{j,\max}$$

where $\theta_{j,\min}$ and $\theta_{j,\max}$ are the lower and upper limits of the angle of the *j*th joint of the SM, respectively.

### 4.1. Coupled Workspace Modeling

- Constrained Workspace of the SM

**Figure 6:**
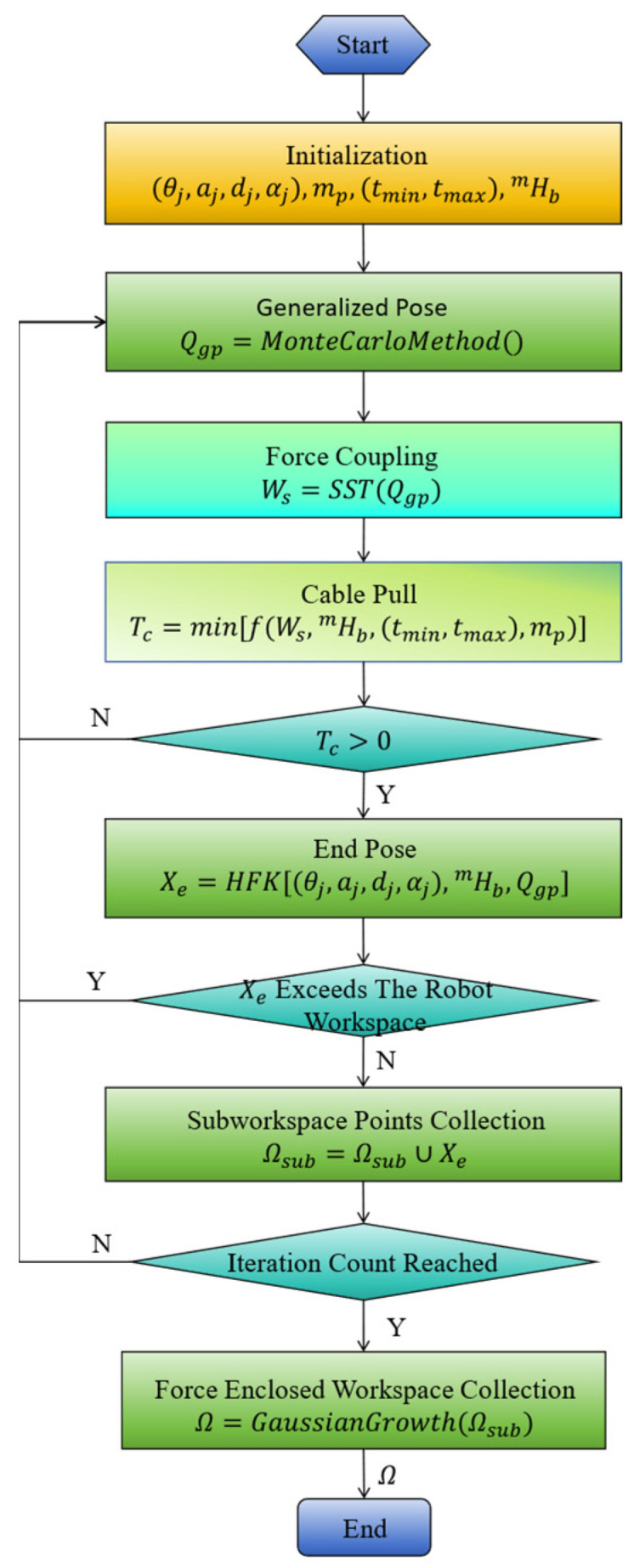
FCW resolving process. SST(·) denotes the recursive Newton–Euler formulation for computing link wrenches of the serial manipulator. HFK[·] denotes the hybrid forward kinematics function of the cable-driven hybrid robot.

The joint angle space of the SM was fully explored when the pose of the moving platform Xmg remained constant. At this point, the set of end-effector pose points of the CDHR form the constrained SM workspace. This reflects the flexibility of SM spatial movement when the CDHR is in a fixed position. Combined Equation (21) with Equation (13), the FCW can be obtained. As shown in Figure 6, random sampling of the joint angles of the SM is performed while satisfying the constraints of Equation (22), and the corresponding generalized pose $Q_{\mathrm{gp}}$ can be expressed as:

(22) $$\begin{array}{c} Q_{\mathrm{gp}}^{\left(k\right)}=f_{\mathrm{random}}\left(Q_{\mathrm{gp},\min},Q_{\mathrm{gp},\max}\right)\\ Q_{\mathrm{gp}}^{\left(k\right)}={\left[x_{m},y_{m},z_{m},\alpha_{m},\beta_{m},\gamma_{m},\theta_{1}^{\left(k\right)},\theta_{2}^{\left(k\right)},\ldots ,\theta_{j}^{\left(k\right)},\ldots ,\theta_{D_{s}}^{\left(k\right)}\right]}^{T} \end{array}$$

where $Q_{\mathrm{gp},\min},\;Q_{\mathrm{gp},\max}$ are the upper and lower bounds of the generalized pose values, respectively, and $f_{\mathrm{random}}\left(\cdot \right)$ represents the set of pose points randomly generated within the generalized pose limits by using the Monte Carlo random method.

Thus, the constrained workspace of the SM $\Omega_{\mathrm{hs}}$ can be described as:

(23) Ωhs=Xek=xek,yek,zek,αek,βek,γek|XmgQgp,min≤Qgp(k)≤Qgp,max

- Constrained Workspace of the SM

**Figure 7:**
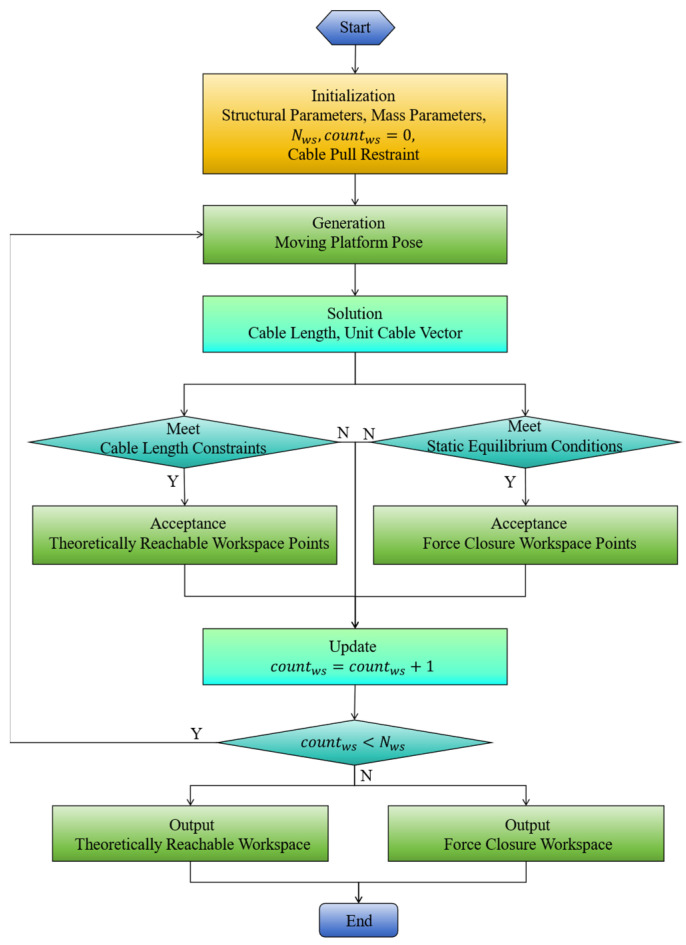
Workspace resolving process of the CDPR.

When the joint angles $\Theta_{s}$ of the SM remained constant, the workspace of the moving platform was fully explored. The set of end-effector pose points of the CDHR constitutes the constrained workspace of the CDPR, reflecting the flexibility of the moving platform’s spatial movement under a fixed load. As shown in Figure 7, by randomly sampling the poses of the moving platform, the corresponding generalized pose $Q_{\mathrm{gp}}$ can be described as:

(24) $$\begin{array}{c} Q_{\mathrm{gp}}^{\left(k\right)}=f_{\mathrm{random}}\left(Q_{\mathrm{gp},\min},Q_{\mathrm{gp},\max}\right)\\ Q_{\mathrm{gp}}^{\left(k\right)}={\left[x_{m}^{\left(k\right)},y_{m}^{\left(k\right)},z_{m}^{\left(k\right)},\alpha_{m}^{\left(k\right)},\beta_{m}^{\left(k\right)},\gamma_{m}^{\left(k\right)},\theta_{1},\theta_{2},\ldots ,\theta_{j},\ldots ,\theta_{D_{s}}\right]}^{T} \end{array}$$

Similarly, the constrained workspace of the CDPR $\Omega_{\mathrm{hp}}$ can be written as:

(25) $$\Omega_{\mathrm{hp}}=\left\{\begin{array}{l} X_{e}^{\left(k\right)}=\left(x_{e}^{\left(k\right)},y_{e}^{\left(k\right)},z_{e}^{\left(k\right)},\alpha_{e}^{\left(k\right)},\beta_{e}^{\left(k\right)},\gamma_{e}^{\left(k\right)}\right)|\Theta_{s},\\ Q_{\mathrm{gp},\min}\leq Q_{\mathrm{gp}}^{\left(k\right)}\leq Q_{\mathrm{gp},\max},\Theta_{\min}\leq \Theta_{s}\leq \Theta_{\max} \end{array}\right\}$$

where $\Theta_{\min}={\left[\theta_{1,\min},\cdots ,\theta_{D_{c},\min}\right]}^{T}$, $\Theta_{\max}={\left[\theta_{1,\max},\cdots ,\theta_{D_{c},\max}\right]}^{T}$.

- Complete FCW

When all generalized joints of the CDHR change, the set of end-effector pose points constitutes the complete FCW. This reflects the flexibility of CDHR when performing tasks in unstructured and constrained environments. As shown in Figure 6, the generalized pose $Q_{\mathrm{gp}}$ is randomly sampled along all dimensions, that is:

(26) $$\begin{array}{c} Q_{\mathrm{gp}}^{\left(k\right)}=f_{\mathrm{random}}\left(Q_{\mathrm{gp},\min},Q_{\mathrm{gp},\max}\right)\\ Q_{\mathrm{gp}}^{\left(k\right)}={\left[x_{m}^{\left(k\right)},y_{m}^{\left(k\right)},z_{m}^{\left(k\right)},\alpha_{m}^{\left(k\right)},\beta_{m}^{\left(k\right)},\gamma_{m}^{\left(k\right)},\theta_{1}^{\left(k\right)},\theta_{2}^{\left(k\right)},\ldots ,\theta_{j}^{\left(k\right)},\ldots ,\theta_{D_{s}}^{\left(k\right)}\right]}^{T} \end{array}$$

The complete FCW set $\Omega_{h}$ is specifically expressed as:

(27) $$\Omega_{h}=\left\{\begin{array}{c} X_{e}^{\left(k\right)}=\left(x_{e}^{\left(k\right)},y_{e}^{\left(k\right)},z_{e}^{\left(k\right)},\alpha_{e}^{\left(k\right)},\beta_{e}^{\left(k\right)},\gamma_{e}^{\left(k\right)}\right)|\Theta_{\min}\leq \Theta_{s}\leq \Theta_{\max},\\ Q_{\mathrm{gp},\min}\leq Q_{\mathrm{gp}}^{\left(k\right)}\leq Q_{\mathrm{gp},\max} \end{array}\right\}$$

### 4.2. Workspace Analysis

- Coupled Workspace Analysis

To describe the workspace boundaries more accurately, the Gaussian enrichment method is used to improve boundary precision. The corresponding simulation parameters are shown in Table 3. $\left(n_{x},n_{y},n_{z}\right)$ is the grid resolution. $\sigma^{\mathrm{ini}}$ is the initial standard deviation; $\omega_{k}$ is the variance-decay factor; $n_{f}^{\max}$ is the maximum number of grid-fill attempts; $N_{c}$ is the maximum number of grid points. The range of joint angles for the SM is provided in Table 4.

**Figure 8:**
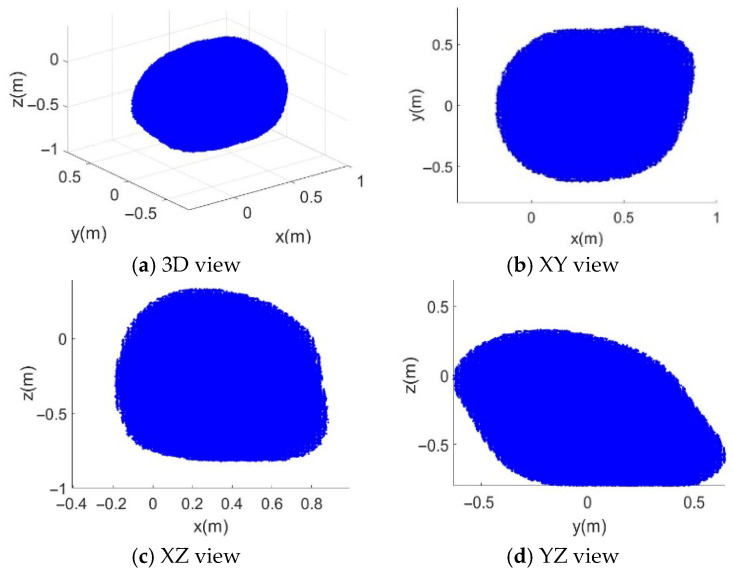
Complete FCW of the CDHR. (**a**) 3D view in (x,y,z); (**b**) projection on the (x,y)-plane; (**c**) projection on the (x,z)-plane; (**d**) projection on the (y,z)-plane. Blue points represent sampled feasible positions satisfying the force-closure condition under the considered constraints.

**Figure 9:**
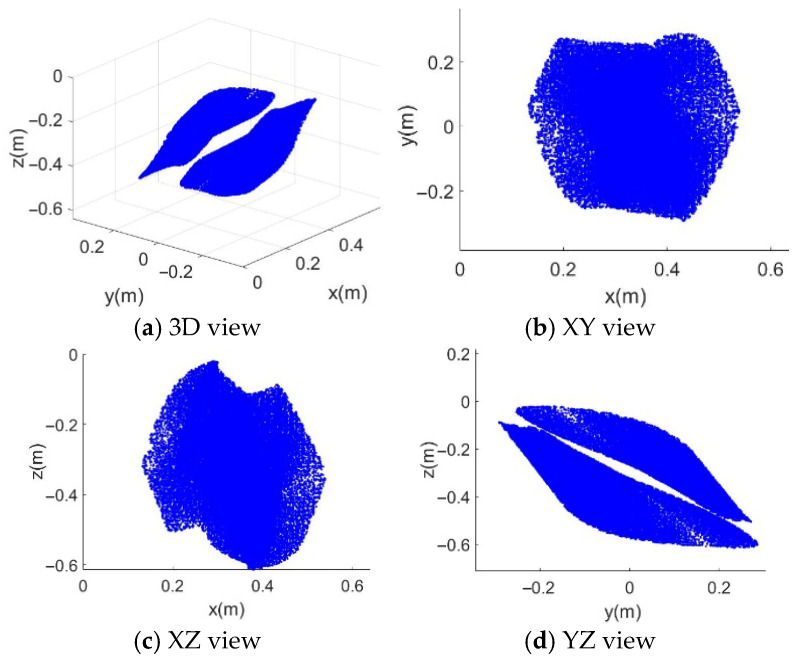
FCW of the CDPR without the SM. (**a**) 3D view in (x,y,z); (**b**) projection on the (x,y)-plane; (**c**) projection on the (x,z)-plane; (**d**) projection on the (y,z)-plane. Blue points represent sampled feasible positions under the same force-closure evaluation.

**Figure 10:**
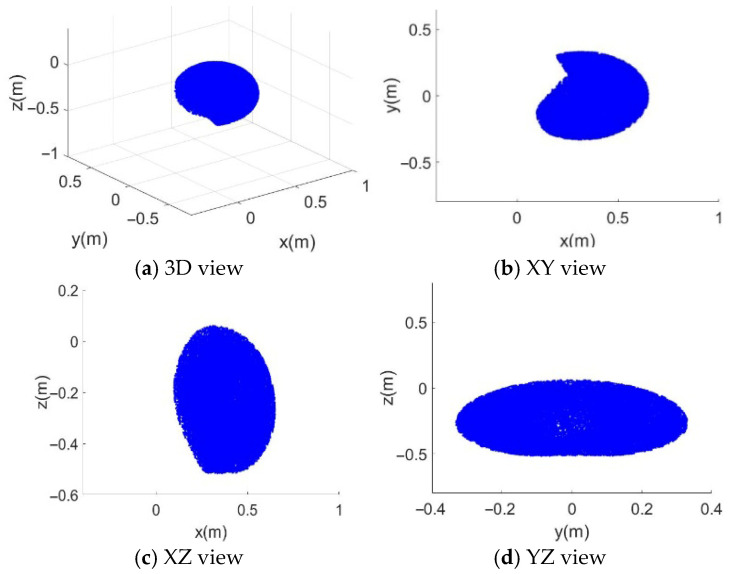
Constrained workspace of the SM with the fixed moving platform. (**a**) 3D view in (x,y,z); (**b**) projection on the (x,y)-plane; (**c**) projection on the (x,z)-plane; (**d**) projection on the (y,z)-plane. Blue points represent reachable end-effector positions under the SM joint-limit constraints.

**Figure 11:**
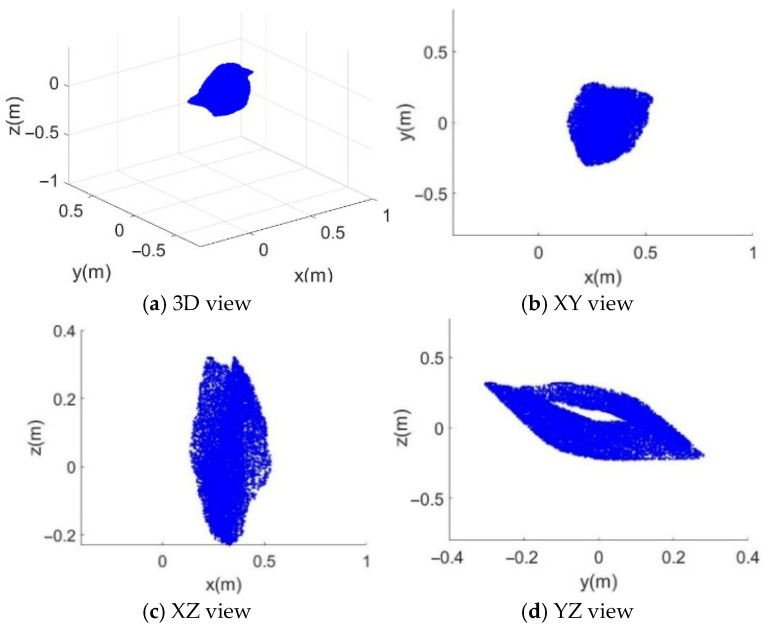
The constrained workspace of the CDPR with the SM pose is fixed. (**a**) 3D view in (x,y,z); (**b**) projection on the (x,y)-plane; (**c**) projection on the (x,z)-plane; (**d**) projection on the (y,z)-plane. Blue points represent sampled feasible platform positions satisfying the force-closure condition with the SM configuration fixed.

In Figure 8, Figure 9, Figure 10 and Figure 11, the blue point cloud denotes the sampled feasible position set (units in meters) that satisfies the corresponding constraints; points outside the feasible set are not plotted. Each figure provides a 3D distribution and its 2D projections on the (XY), (XZ), and (YZ) planes to visualize the boundary. Comparing Figure 8 with Figure 9, the feasible set becomes larger when the serial manipulator is included, especially along the arm extension direction, and feasibility is recovered in regions where the CDPR-only case violates the force-closure condition. The CDPR does not satisfy the force-closure condition at some points near the center, whereas the CDHR remains reachable in these regions.

When the moving platform pose is set as $X_{m}={\left[0.32,0,-0.32,0,0,0\right]}^{T}$, the generated constrained workspace of the SM is shown in Figure 10. The pose of the moving platform was near the center of the space formed by the aluminum frame of the robot. In each dimension, the geometric shape of the robot end-effector workspace boundary formed a slightly imperfect ellipse. The missing portions were caused by the joint angle limitations of the SM, indicating that the workspace loss owing to structural coupling was minimal.

With the initial joint angles of SM set to $\Theta_{s}={\left[0,\pi /2,0,0\right]}^{T}$, the constrained workspace of the CDPR is shown in Figure 11. From Figure 11c,d, it can be observed that the reachable positions along the z-axis are located outside the convex hull formed by the fixed anchor points. This is because the manipulator is mounted on the upper surface of the moving platform, causing the constrained workspace of the CDPR to shift positively along the z-axis. Figure 11b,c show that along the x- and y-axes, the reachable workspace is significantly reduced compared with the complete FCW.

- Workspace Analysis of Arm Flexibility

According to Equation (18), when the joint angles of the SM change, the force exerted by the SM on the moving platform remains constant but the applied moment changes. Therefore, when the pose of the moving platform remains unchanged and the configuration of the SM changes, the static equilibrium equation of the moving platform can be expressed by Equation (20) may not yield a numerical solution. Thus, it is necessary to determine the arm flexibility of the CDHR and evaluate the quality of the robot workspace. Using the Monte Carlo method, the joint angles $\Theta_{s}$ of the SM can be randomly generated According to Equation (9), the relative pose Xeb from the SM base to the end effector can be calculated. By substituting Xeb and $\Theta_{s}$ into Equation (28) and repeating the process times yields the corresponding subspace as follows:

(28) Ωm:Xeκ|Xeκb=CFKpbi,rai,Tbm,Qgjκ,κ∈1,2,…,NaΩs:Qgjκ=θ1κ,θ2κ,…,θDsκ,u∈1,2,…,Na

According to Equation (28), three different sectional equations are selected to compute the flexibility space:

(29) $$\begin{array}{c} \Phi_{1}:\left\{\begin{array}{l} x=x_{\min}+i_{x}\cdot \frac{\left(x_{\max}-x_{\min}\right)}{50}\\ y=y_{\min}+i_{y}\cdot \frac{\left(y_{\max}-y_{\min}\right)}{50}\\ z=z_{\min}+\frac{0.5i_{x}}{\left(z_{\max}-z_{\min}\right)}+\frac{0.5i_{y}}{\left(z_{\max}-z_{\min}\right)} \end{array}\right.,\Phi_{2}:\left\{\begin{array}{l} x=0.32\\ y=y_{\min}+i_{y}\cdot \frac{\left(y_{\max}-y_{\min}\right)}{50}\\ z=z_{\min}+i_{z}\cdot \frac{\left(z_{\max}-z_{\min}\right)}{50} \end{array}\right.\\ \Phi_{3}:\left\{\begin{array}{l} x=x_{\min}+i_{x}\cdot \frac{\left(x_{\max}-x_{\min}\right)}{50}\\ y=y_{\min}+i_{y}\cdot \frac{\left(y_{\max}-y_{\min}\right)}{50}\\ z=-0.32 \end{array}\right. \end{array}$$

where $z_{\max}$ = 0.024 m, $z_{\min}$ = −0.626 m, $y_{\max}$ = 0.385 m, $y_{\min}$ = −0.385 m, $x_{\max}$ = 0.645 m, $x_{\min}$ = 0.025 m, the values for $i_{x}$, $i_{y}$ and $i_{z}$ range from 0,1,2,3,⋯,50.

**Figure 12:**
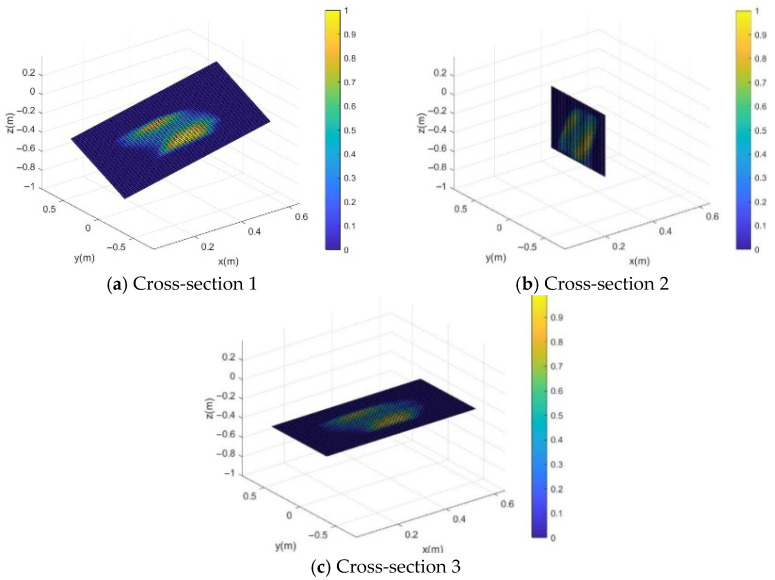
Heat maps of the CDHR’s flexibility at three different cross-sections. (**a**) represents cross-section $\Phi_{1}$, (**b**) represents cross-section $\Phi_{2}$, and (**c**) represents cross-section $\Phi_{3}$. The color spectrum represents the distribution of flexibility values, where yellow indicates greater flexibility, and blue indicates lower flexibility.

As shown in Figure 12, the CDHR satisfies the force-closure condition near the central region of the bounding box. However, at the edges of the workspace, the flexibility of the CDHR decreases significantly.

## 5. Dynamic Anchor Seat Position Optimization Method Based on the Global Condition Number

The direction of cable tension on the moving platform of the CDHR is determined by the positions of the anchor seats on the housing and moving platform. To ensure movement flexibility, the positions of the anchor seats on the moving platform need to be optimized. Owing to the unidirectional nature of cable forces, the cable tension applied to the moving platform can form a convex hull surrounding the platform’s center of mass, providing force/torque in any direction force [17]. The global condition number is an important indicator for evaluating the flexibility and performance of the robot [34]. Based on this, this study optimizes the shape and installation pose of the dynamic anchor seats on the prototype using the global condition number.

The static equilibrium equation of the moving platform in the CDHR can be expressed as a system of linear equations, i.e., Ax=b, $A=A_{p}$, $x=T_{c}$, $b=-G_{p}-W_{\mathrm{em}}$. The solution to this system of linear equations represents the magnitude of cable tension. When the external forces change, the generalized force $W_{\mathrm{em}}$ applied by the SM on the moving platform will also change. The condition number is used to measure the sensitivity of x to changes in b in the linear system Ax=b. The smaller the condition number, the less impact perturbations in b have on x. Therefore, the condition number can be described as:

(30) $$\kappa \left(A\right)=\mathrm{cond}(A)={\left\|A\right\|}_{2}\cdot {\left\|A^{-1}\right\|}_{2}=\frac{\sigma_{\max}\left(A\right)}{\sigma_{\min}\left(A\right)}$$

where $\kappa \left(A\right)\in \left[1,\infty \right]$ is the condition number of matrix A, ${\left\|A\right\|}_{2}$ is the 2-norm of matrix A, $\sigma_{\max}\left(A\right)$ is the largest singular value of matrix A, and $\sigma_{\min}\left(A\right)$ is the smallest singular value of matrix A.

The global condition numbers defined in Equation (30) can be used to describe the degree to which the motion performance of the hybrid robot approaches isotropy at different poses [22], i.e.,

(31) $$\mathrm{GCI}=\frac{\int_{U}\kappa {\left(A\right)}^{-1}du}{\int_{U}du}$$

where $n_{\kappa }$ is the size of the discrete set of workspace points for the CDHR.

The influence of the cable forces on the moving platform in each motion dimension can be represented by the structure matrix $A_{p}$ of the CDHR. As $A_{p}$ contains both translational and rotational components, the structure matrix $A_{p}$ can be normalized to maintain consistent motion performance along the principal axes of each dimension [24]. The normalized structure matrix can be expressed as:

(32) $${\hat{A}}_{p}=\left[\begin{array}{cccc} {\hat{L}}_{1} & {\hat{L}}_{2} & \ldots & {\hat{L}}_{D_{c}}\\ \frac{r_{a1}\times {\hat{L}}_{1}}{L_{\mathrm{ch}}} & \frac{r_{a2}\times {\hat{L}}_{2}}{L_{\mathrm{ch}}} & \ldots & \frac{r_{aD_{c}}\times {\hat{L}}_{D_{c}}}{L_{\mathrm{ch}}} \end{array}\right]$$

where $L_{\mathrm{ch}}$ is the characteristic length of the matrix $A_{p}$.

Based on Equation (33), the corresponding motion flexibility in this state can be obtained as:

(33) $$\begin{array}{l} M_{q}=\mathrm{diag}\left({\hat{A}}_{p}\cdot {\hat{A}}_{p}^{T}\right)\\ =\mathrm{diag}\left(\left[\begin{array}{cc} \sum_{i=1}^{D_{c}}{\hat{L}}_{i}\cdot {\hat{L}}_{i}^{T} & \sum_{i=1}^{D_{c}}\frac{{\hat{L}}_{i}\cdot {\left(r_{ai}\times {\hat{L}}_{i}\right)}^{T}}{L_{\mathrm{ch}}}\\ \sum_{i=1}^{D_{c}}\frac{\left(r_{ai}\times {\hat{L}}_{i}\right)\cdot {\hat{L}}_{i}^{T}}{L_{\mathrm{ch}}} & \sum_{i=1}^{D_{c}}\frac{\left(r_{ai}\times {\hat{L}}_{i}\right)\cdot {\left(r_{ai}\times {\hat{L}}_{i}\right)}^{T}}{L_{\mathrm{ch}}} \end{array}\right]\right) \end{array}$$

Assuming that the CDHR satisfies the condition of isotropic motion, i.e., $M_{q}=\sigma^{2}E_{6}$, where $E_{6}$ is the 6-dimensional identity matrix, according to Equation (34), we obtain:

(34) $$\left\{\begin{array}{l} \sum_{i=1}^{D_{c}}{{\hat{L}}_{i,x}}^{2}=\sum_{i=1}^{D_{c}}{{\hat{L}}_{i,y}}^{2}=\sum_{i=1}^{D_{c}}{{\hat{L}}_{i,z}}^{2}=\sigma^{2}\\ \sum_{i=1}^{D_{c}}\frac{{\left(r_{ai,y}^{2}\cdot {\hat{L}}_{i,z}-r_{ai,z}^{2}\cdot {\hat{L}}_{i,y}\right)}^{2}}{L_{\mathrm{ch}}^{2}}=\sigma^{2}\\ \sum_{i=1}^{D_{c}}\frac{{\left(r_{ai,z}^{2}\cdot {\hat{L}}_{i,x}-r_{ai,x}^{2}\cdot {\hat{L}}_{i,z}\right)}^{2}}{L_{\mathrm{ch}}^{2}}=\sigma^{2}\\ \sum_{i=1}^{D_{c}}\frac{{\left(r_{ai,x}^{2}\cdot {\hat{L}}_{i,y}-r_{ai,y}^{2}\cdot {\hat{L}}_{i,z}\right)}^{2}}{L_{\mathrm{ch}}^{2}}=\sigma^{2} \end{array}\right.$$

where $\left({\hat{L}}_{i,x},{\hat{L}}_{i,y},{\hat{L}}_{i,z}\right)$ are the components of ${\hat{L}}_{i}$ along each coordinate axis, and $\left(r_{ai,x},r_{ai,y},r_{ai,z}\right)$ are the components of $r_{ai}$ along each coordinate axis. By combining the subexpressions in Equation (35), the characteristic length of matrix ${\hat{A}}_{p}$ can be obtained as:

(35) $$L_{\mathrm{ch}}=\sqrt{\frac{{\left(L_{chx}\right)}^{2}+{\left(L_{chy}\right)}^{2}+{\left(L_{chz}\right)}^{2}}{D_{c}}}=\frac{\left\|r_{ai}\times {\hat{L}}_{i}\right\|}{D_{c}}$$

where $L_{chx}=r_{ai,y}^{2}\cdot {\hat{L}}_{i,z}-r_{ai,z}^{2}\cdot {\hat{L}}_{i,y}$, $L_{chy}=r_{ai,z}^{2}\cdot {\hat{L}}_{i,x}-r_{ai,x}^{2}\cdot {\hat{L}}_{i,z}$, $L_{chz}=r_{ai,x}^{2}\cdot {\hat{L}}_{i,y}-r_{ai,y}^{2}\cdot {\hat{L}}_{i,z}$.

By substituting Equation (36) into Equation (33), the structural matrix of the CDHR can be obtained. Combining this with Equations (30) and (32), the condition number and global condition number of ${\hat{A}}_{p}$ are given as:

(36) $$\kappa \left({\hat{A}}_{p}\right)=\frac{\sigma_{\max}\left({\hat{A}}_{p}\right)}{\sigma_{\min}\left({\hat{A}}_{p}\right)}$$

where $N_{\kappa }$ represents the number of discrete pose points in the set $\Omega_{m}$.

To ensure that the global condition number in the discrete case closely approximated the actual global condition number, the reachable workspace $\Omega_{m}$ of the moving platform was uniformly discretized. Thus, the position optimization model for the moving platform anchor seats can be expressed as:

(37) $$X_{ra}=\arg\min\left(1-\sum_{k=1}^{n_{\kappa }}\kappa {\left({\hat{A}}_{p}^{\left(k\right)}\right)}^{-1}/n_{\kappa }\right)$$

Considering that a spherical distribution of anchor seats balances forces in all directions and reduces structural deformation of the moving platform caused by uneven cable tensions, we adopt a moving platform with anchors arranged on a sphere in this study. Let $d_{a}$ be the radius of the sphere with $d_{a}$ = 75 mm. Let $\theta_{ai}$ be the angle between the line segment connecting the origin of the moving coordinate system to the anchor seat and the z-axis of the moving coordinate system and be the angle between the projection of this line segment onto the XY plane and the x-axis of the moving coordinate system. Based on the correspondence between the spherical and Cartesian coordinates, the spherical coordinates of the anchor seat can be expressed as

(38) $$\left\{\begin{array}{l} r_{ai,x}=d_{a}\cdot \sin\theta_{ai}\cdot \cos\varphi_{ai}\\ r_{ai,y}=d_{a}\cdot \sin\theta_{ai}\cdot \sin\varphi_{ai}\\ r_{ai,z}=d_{a}\cdot \cos\theta_{ai} \end{array}\right.$$

Furthermore, the optimization variables can be designed to $X_{ra}={\left[d_{a},\theta_{a1},\varphi_{a1},\ldots ,\theta_{aD_{c}},\varphi_{aD_{c}}\right]}^{T}$, reduce the dimensionality from 1 + 2$D_{c}$ by $D_{c}$ − 1. The optimization objective corresponding to the global condition number is:

(39) $$\begin{array}{l} \min f\left(X_{ra}\right)\\ s.t.\;X_{e}\in \Omega_{h} \end{array}$$

**Figure 13:**
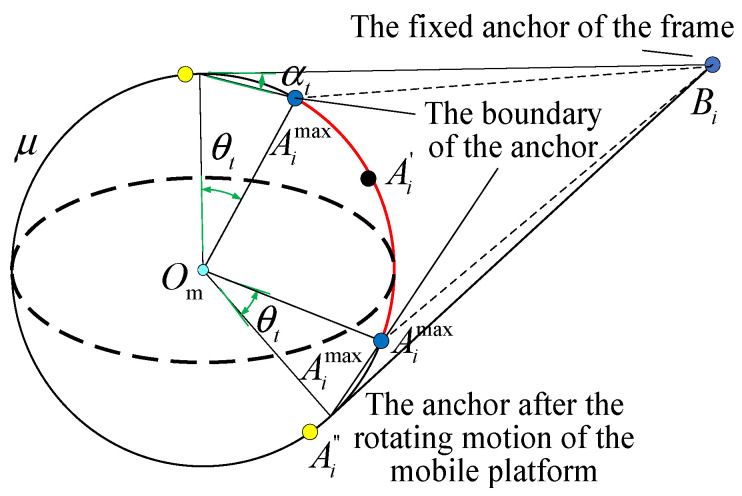
Intersection collision analysis of the sphere center–moving anchor–fixed anchor plane.

In this study, the Particle Swarm Optimization (PSO) algorithm is used to solve Equation (41). Because the line segments connecting the fixed anchor points and the moving anchor points may collide with the moving platform, it is necessary to penalize the fitness of particles to remove these unreasonable moving anchor points. As shown in Figure 13, a collision analysis is conducted on the plane μ formed by the sphere center, a single moving anchor seat, and a single fixed anchor seat. Let $\theta_{t}$ represent the reserved tolerance rotation angle, and $A_{i}^{\prime \prime }$ represent the tolerance deviation angle mapped to the tangent. $A_{i}^{\prime \prime }$ represents the position of the moving platform anchor seat during optimization, and represents the position of the moving anchor seat corresponding to the maximum rotation angle of the moving platform around the normal vector of the plane μ. $A_{i}^{\max}$ represents the position boundary of the moving platform anchor seat during optimization. Based on geometric relationships, $\alpha_{t}=\theta_{t}/2$. The red arc represents the safe region of anchor seat distribution in the tangent plane. The safe region in the three-dimensional space for the moving platform anchor seat is the surface generated by rotating the red arc around the plane μ, which connects the fixed anchor seat $O_{m}B_{i}$ by 360°.

According to the above analysis, the evaluation score for intersection collision $s_{1}$ can be expressed as:

(40) $$s_{1}=\frac{A_{i}^{\prime }B_{i}-A_{i}^{\max}B_{i}}{O_{m}B_{i}}$$

where $A_{i}^{\prime \prime }A_{i}^{\max}=\sqrt{2\cdot d_{a}^{2}\left(1-\cos\left(\theta_{t}\right)\right)}$, $A_{i}^{\max}B_{i}=\sqrt{{\left(A_{i}^{\prime \prime }B_{i}\right)}^{2}+{\left(A_{i}^{\prime \prime }A_{i}^{\max}\right)}^{2}-2A_{i}^{\prime \prime }B_{i}\cdot A_{i}^{\prime \prime }A_{i}^{\max}\cdot \cos\left(\alpha_{t}\right)}$.

Thus, the particle fitness $\upsilon_{f}$ in the PSO algorithm can be expressed as:

(41) $$\upsilon_{f}=\left\{\begin{array}{l} 1+s_{1},\;\mathrm{If}\;\mathrm{there}\;\mathrm{is}\;a\;\mathrm{cross}\;\mathrm{collision}\\ s_{2},\;\mathrm{else} \end{array}\right.$$

where the evaluation score of the particle $s_{2}=1-\sum_{k=1}^{n_{\kappa }}\kappa {\left({\hat{A}}_{p}\right)}^{-1}/n_{\kappa }$.

## 6. Vision-Based Self-Calibration Method for Kinematic Parameters

### 6.1. Multi-Mapping Calibration Problem of the Hybrid Robot

**Figure 14:**
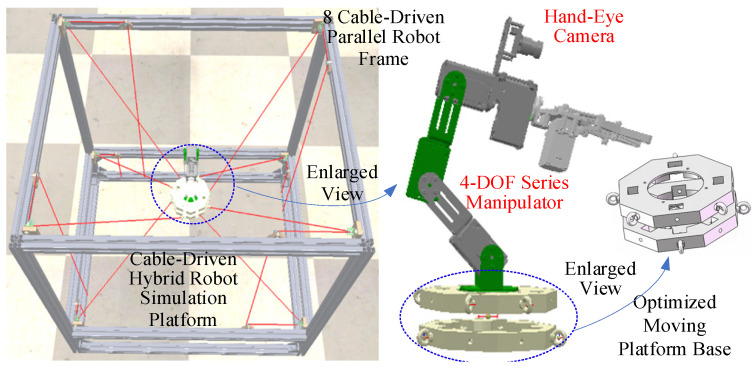
Simulation model of the CDHR.

As shown in Figure 14, when deploying the reconfigurable CDHR, it is necessary to calibrate the positions of the fixed anchor seats, installation position of the SM, and the hand-eye relationship. Typically, the coordinates of moving anchor seats relative to the moving coordinate system $r_{ai}$ are known. The global frame {G} is established on the April QR code [35] and aligned with the April frame. {V} represents the camera frame mounted on the robot, {E} is the coordinate system of the SM end-effector, and {T} is the frame of the collaboration marker. After installing the CDHR, the motor encoder count is reset to zero at the initial moment. During the actual operation, the change in the cable length was measured using angle sensors and converted (as in Equation (4)), yielding the current cable length **l**. Next, the April QR code recognition algorithm calculates the HTM (i.e., Tg.v) from the QR code frame to the camera frame. The joint angles $\Theta_{s}$ of the SM are obtained via feedback from the joint servomotors, allowing the calculation of $Q_{\mathrm{gj}}$. Thus, given $N_{\mathrm{calib}}$ sets of generalized joint measurements $Q_{\mathrm{gj}}$, it is possible to simultaneously calibrate the position of the fixed anchor seats $p_{bi}$, the relative pose between the moving platform and base, and the hand-eye external parameter matrix of the end-effector.

### 6.2. Resolving of the Self-Calibration Equation for the CDHR

In the global frame, the position vector of the moving anchor seat can be expressed as:

(42) raig=tvg+Rvg⋅tev+Reg⋅tbe+Rbg⋅tmb+Rmg⋅rim=tvg+Rvg⋅tev+Rvg⋅Rev⋅tbe+Rvg⋅Rev⋅Rbe⋅tpb+Rvg⋅Rev⋅Rbe⋅Rmb⋅raim

where Tdc represents the HTM from frame {c} to frame {d}, Rdc and tdc represent the rotation matrix and translation vector of Tdc, respectively. These satisfy the conditions Rdc=Tdc1:3,1:3=Tcd1:3,1:3−1, tdc=Tdc1:3,4=Tcd1:3,4−1.

Furthermore, the actual length of the *i*th cable $l_{i}$ can be expressed as:

(43) li=fcalibpbig,raim,Xmb,Xev,Tgv,Teb=pbig−raig2

where Xdc represents the relative pose of frame {d} with respect to frame {c}. Xmb represents the relative pose of moving platform {m} with respect to base {b}. X˜m0b is the initial value of Xmb.

The solution to Equation (45) can be obtained using the least squares method. Let the iterative values of Xmb, Xev, and pbig be X˜mb, X˜ev, and p˜big, respectively. Then, the error model for the i-th cable is:

(44) Xmb,Xev,pbig=argmin∑iDcl^i−li˜2s.t. li˜=fcalibp˜big,raim,X˜mb,X˜ev,Tgv,Teb   i=1,2,…,Dc

where ${\hat{l}}_{i}$ is the actual value measured by the drum rotation angle sensor.

For a CDHR with $D_{c}$ cables, each set of poses can establish $D_{c}$ equations as shown in Equation (45). The unknown calibration variables, Xmb, Xev and pbig, include a total of $12+3D_{c}$ unknowns. To fully solve this calibration problem, data from at least $\mathrm{ceil}\left(\frac{12+3D_{c}}{D_{c}}\right)$ different poses is required, where $\mathrm{ceil}\left(\cdot \right)$ represents the ceiling function. Here, we select $N_{\mathrm{calib}}>\mathrm{ceil}\left(\frac{12+3D_{c}}{D_{c}}\right)$.

Let the superscript k denote the group index of the calibration data. The synchronous calibration optimization function for the $N_{\mathrm{calib}}$ sets of data is:

(45) Xmb,Xev,pbig=argmin∑kNcalib∑iDcl^ik−lik˜2s.t. lik˜=fcalibp˜big,raim,X˜mb,X˜ev,Tgkv,Tekbi=1,2,⋯,Dc, k=1,2,…,Ncalib

where ${\hat{l}}_{i}^{\left(k\right)}$, $\tilde{l_{i}^{\left(k\right)}}$ are measured and calculated lengths of the *i*th cable in the *k*th set of data, respectively.

The overall algorithm process is shown in Algorithm 1. By selecting appropriate initial values for the iterations, the convergence speed of the optimization algorithm can be improved. When the SM is installed at the center of the moving platform and the base frame aligns with the axes of the moving coordinate system, the initial values X˜m0b can be chosen as ${\left[0,0,\frac{h_{m}}{2},0,0,0\right]}^{T}$ or ${\left[0,0,-\frac{h_{m}}{2},0,0,0\right]}^{T}$, where $h_{m}$ represents the height of the moving platform.
**Algorithm 1:** Kinematic Parameter Self-Calibration Algorithm for Cable-Driven Hybrid Robots    **Input:** Structural height $h_{m}$, measured cable length set $\left\{{\hat{l}}_{i}^{\left(k\right)}\right\}$, convergence threshold ϵ, $N_{c}$    **Output:** Calibrated parameters Xmb,Xev,pbig.    **Initialization:** Initialize parameter vector Θ(0)=[X˜m(0)b,X˜e(0)v,p˜bi(0)g], with X˜m(0)b$\;\mathrm{set}\;\mathrm{to}{\left[0,0,\pm h_{m}/2,0,0,0\right]}^{T}$.    **Data Collection:** Collect cable length data for $N_{calib}$ poses, satisfying $N_{calib}>\mathrm{floor}((12+3D_{c})/D_{c})$.    $\mathrm{While}\;t<N_{max}$ and error >ϵ do:        $\mathrm{Calculate}\;\mathrm{theoretical}\;\mathrm{cable}\;\mathrm{lengths}\;{\tilde{l}}_{i}^{\left(k\right)}$ using Equation (43) with current parameters.        Construct the least-squares objective function: $\min\sum_{k}\sum_{i}\left\|{\hat{l}}_{i}^{\left(k\right)}-{\tilde{l}}_{i}^{\left(k\right)}\right\|$.        $\mathrm{Update}\;\mathrm{parameter}\;\mathrm{vector}\;\Theta^{\left(t+1\right)}$ using the Levenberg–Marquardt (LM) algorithm.        Calculate residuals and check for convergence.    End  Return Optimized parameter set $\Theta^{*}$.

## 7. Model Predictive Control-Based Trajectory Tracking Method

The model of the CDHR in the generalized discrete joint space at time step k can be expressed as:

(46) $$q_{k+1}=Aq_{k}+Bu_{k}$$

Let

(47) $$\begin{array}{l} \delta q_{k}=q_{k}-q_{k-1}\\ \delta u_{k}=u_{k}-u_{k-1} \end{array}$$

Let ${\hat{q}}_{k}^{d}$ be the reference generalized joint position and $q_{k}^{d}$ the desired generalized joint position. The tracking error between the actual generalized joint position and the reference generalized joint position at time step k is defined as $e_{k}=q_{k}-{\hat{q}}_{k}^{d}$. The system state variable is $\xi_{k}={\left[\delta {q_{k}}^{T},{e_{k}}^{T}\right]}^{T}$. Thus, the dynamic augmented system equation can be expressed as:

(48) $$\xi_{k+1}=\left[\begin{array}{cc} A & O_{D_{h}}\\ A & I_{D_{h}} \end{array}\right]\xi_{k}+\left[\begin{array}{c} B\\ B \end{array}\right]\delta u_{k}$$

where $q=Q_{gj}\in {\mathbb{R}}^{D_{h}}$ represents the generalized joint positions of the CDHR, $A=I_{D_{h}}\in {\mathbb{R}}^{D_{h}\times D_{h}}$ is the identity state matrix, $B=I_{D_{h}}\Delta t\in {\mathbb{R}}^{D_{h}\times D_{h}}$ is the control matrix, and Δt represents the discrete time interval. The control input $u=\dot{q}$ denotes the generalized joint velocities of the CDHR.

Let $\hat{A}=\left[\begin{array}{cc} A & O_{D_{h}}\\ A & I_{D_{h}} \end{array}\right]$, $\hat{B}=\left[\begin{array}{c} B\\ B \end{array}\right]$, the tracking model for the reference trajectory can be designed as:

(49) $$\begin{array}{l} V_{M}^{O*}(\xi_{k},q_{k}^{d})=\underset{\delta U_{k},{\hat{q}}_{k}}{\min}L_{p}\\ s.t.\;\left\{\begin{array}{l} \xi_{k+i+1}=\hat{A}\xi_{k+i}+\hat{B}\delta u_{k+i}\\ \delta u_{k+i}\in U\\ \left[\begin{array}{c} q_{k}\\ u_{k-1} \end{array}\right]\in \mathbb{Q}\times U\\ \zeta_{k+M}\in O \end{array}\right. \end{array}$$

where $L_{p}=\sum_{i=0}^{M-1}\left({\left\|\xi_{k+i}\right\|}_{Q_{1}}^{2}+{\left\|\delta u_{k+i}\right\|}_{Q_{2}}^{2}\right)+{\left\|\xi_{k+M}\right\|}_{Q_{3}}^{2}+{\left\|{\hat{q}}_{k}-q_{k}^{d}\right\|}_{Q_{4}}^{2}$, $\delta U_{k}={\left[\delta u_{k}^{T},\delta u_{k+1}^{T},\ldots ,\delta u_{k+M-1}^{T}\right]}^{T}$ represents the incremental control sequence defined over the horizon M. $Q_{1}\in {\mathbb{R}}^{2D_{h}\times 2D_{h}}$, $Q_{2}\in {\mathbb{R}}^{2D_{h}\times 2D_{h}}$, $Q_{3}\in {\mathbb{R}}^{2D_{h}\times 2D_{h}}$ and $Q_{4}\in {\mathbb{R}}^{2D_{h}\times 2D_{h}}$ are positive definite weighting matrices. $\zeta_{k}$ is the convex polyhedron constraint set, and $\zeta_{k}={\left[\xi_{k}^{T},{\hat{q}}_{k}^{T}\right]}^{T}$, where O represents the Maximal Output Admissible Set (MOAS).

## 8. Simulation and Experiment Study

### 8.1. Simulation Study

#### 8.1.1. Simulation System Setup

To validate the methods proposed earlier, a simulation system for the CDHR was built. In the simulation, the parameters are set as follows: R_r_ = 6 mm, m_p_ = 0.259 kg, t_min_ = 5 N and t_max_ = 50 N. The DH parameters of the SM are listed in Table 5, where the masses of links 1, 2, 3, and 4 were 0.1290, 0.2240, 0.2240, and 0.4695 kg, respectively.).

#### 8.1.2. Numerical Optimization of the Dynamic Anchor Seat Position

**Figure 15:**
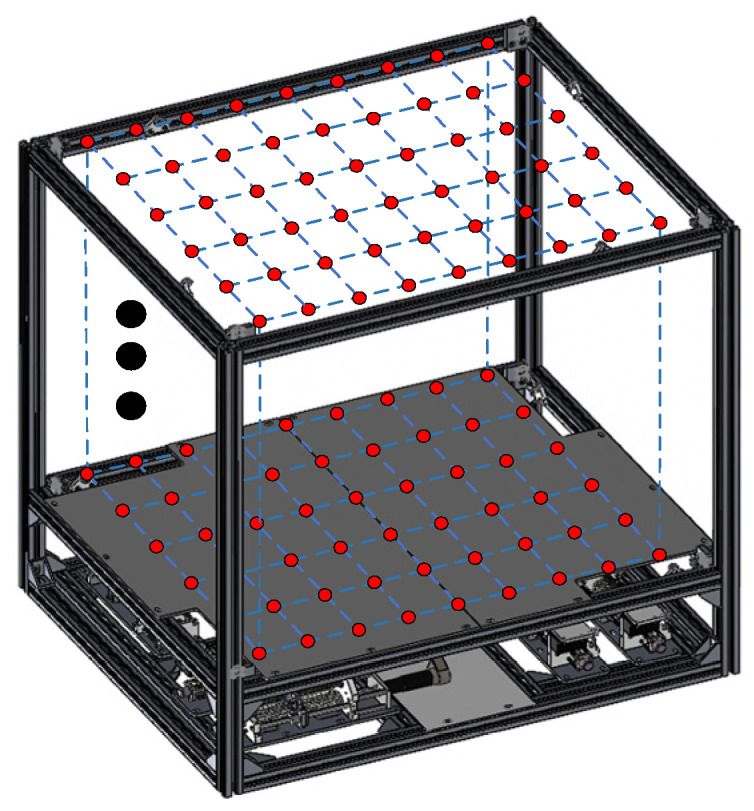
Illustration of uniformly discretized sampled pose points.

The reachable workspace of the CDHR is uniformly discretized. The parameter values are listed in Table 6, and the scatter plot after discretization is shown in Figure 15.

**Figure 16:**
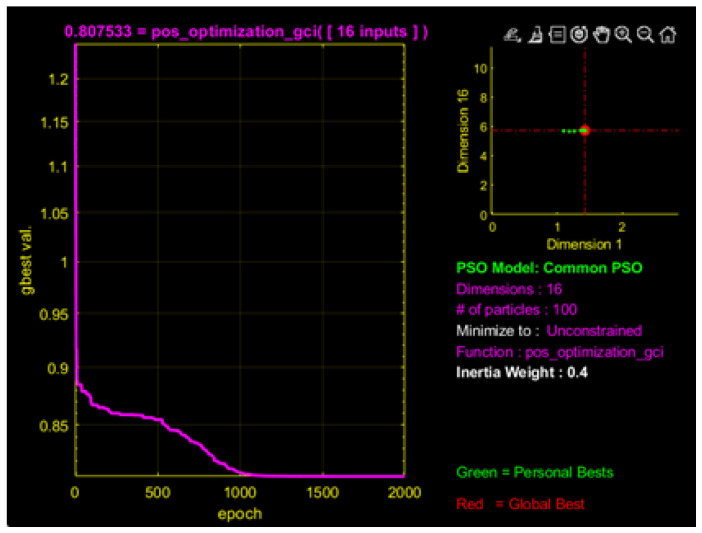
Optimization interface for dynamic anchor seat pose based on the PSO method.

**Figure 17:**
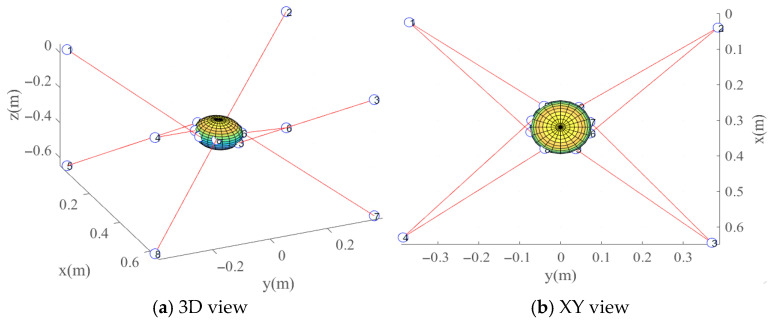
Visualization of the optimized distribution.

The specific parameters used in the PSO iterations are listed in Table 7. The optimization results are shown in Figure 16 based on the model described in Section 5. After 2000 iterations, the objective function value reached 0.8075, corresponding to a global condition number of 0.1925, indicating a significant improvement in the flexibility of the CDHR. The distribution of the optimized dynamic platform anchor seats is listed in Table 8, and the optimized point distribution is shown in Figure 17.

From the illustration of the optimization results, the distribution of the eight dynamic anchor seats resembles a regular octagon. When the anchor seats are arranged in this shape on a horizontal cross-section, the mobility of the moving platform is maximized, and the probability of collisions between the cables and the platform is minimized. The designed distribution of the anchor seats on the moving platform was symmetric with respect to the platform’s center of mass, with square holes left in both the upper and lower layers to accommodate the wiring of the SM motors. Eye bolts were installed on the sides of the moving platform, with the center of each eyebolt ring corresponding to the location of the dynamic anchor seat. The eye bolts were secured with nuts, and the force direction of the eyebolts was aligned with the direction of the cable. The fixation method is non-tightening, allowing the eyebolts to rotate axially to some extent under the cable tension.

#### 8.1.3. Simulation of Self-Calibration of Structural Parameters

In this simulation, $N_{\mathrm{calib}}$ = 30, X˜m0b=0,0,0,0,0,−πT, X˜e0v=0,0,0,0,0,0T. The error between the measured and true values can be described as:

(50) $$\Delta X={\left\|\left(\cdot \right)-\tilde{\left(\cdot \right)}\right\|}_{2}$$

where $\left(\cdot \right)$ and $\tilde{\left(\cdot \right)}$ represent the true and calibrated values of the unknowns, respectively.

As shown in Table 7, these errors are at the sub-millimeter and sub-tenth-degree level (max. 0.10 mm and 0.07°), which indicates that the proposed self-calibration can provide an accurate geometric mapping for subsequent planning and control. Considering the prototype scale (workspace on the order of hundreds of millimeters), the residual positioning error corresponds to a relative error well below 0.1%, and thus the calibration accuracy is sufficient for the intended manipulation and tracking tasks.

#### 8.1.4. Simulation of Trajectory Tracking

To verify the established model, three typical trajectories were simulated and analyzed. The specific parameters of the simulations are presented in Table 8, and the results are shown in Table 9.

**Figure 18:**
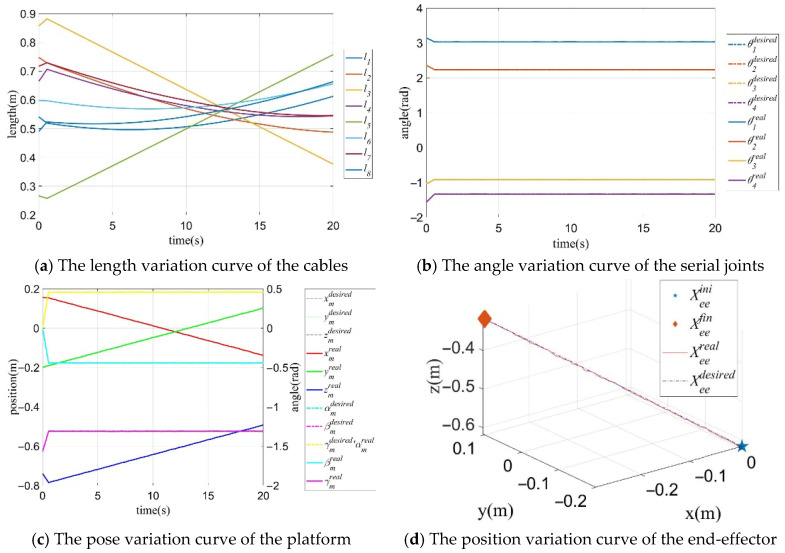
Trajectory variation curves of the mobile platform pose, generalized joints and the end-effector with Case A.

The initial pose is chosen as a representative central feasible pose (inside the FCW) to avoid boundary effects and ensure all constraints are satisfied at the start of tracking. Assume that the initial generalized pose is $Q_{\mathrm{gp}}^{\left(0\right)}$ = [0.1550, −0.2000, −0.7400, 0, −1.5701, 0, 3.14159274, 2.3561945, −1.04719758, −1.57079637]^T^, $\lambda_{1}$ = 1, $\lambda_{2}$ = 1, the number of sampling points is $N_{i}$ = 50,000. The pose variation trajectory of the mobile platform during the linear trajectory tracking process is shown in Figure 18a,b, the trajectory curve of the generalized joint is shown in Figure 18c, and the motion trajectory of the tips of the CDHR is shown in Figure 18d. From the simulation results, it can be observed that the mobile platform produces only a small deflection to avoid generating a large cable tension.

**Figure 19:**
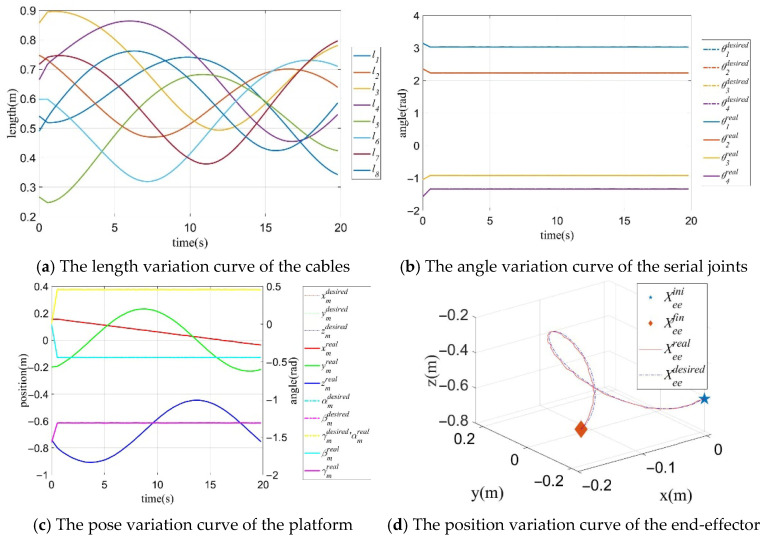
Trajectory variation curves of the mobile platform pose, generalized joints and the end-effector with Case B.

In the spiral trajectory tracking process, the pose variation curves of the mobile platform are shown in Figure 19a,b, the variation curves of the generalized joint are shown in Figure 19c, and the comparison results of the actual and desired trajectories of the CDHR are shown in Figure 19d. Figure 19 shows that the poses of the mobile platform and arm shape of the RSR are motional complementary.

**Figure 20:**
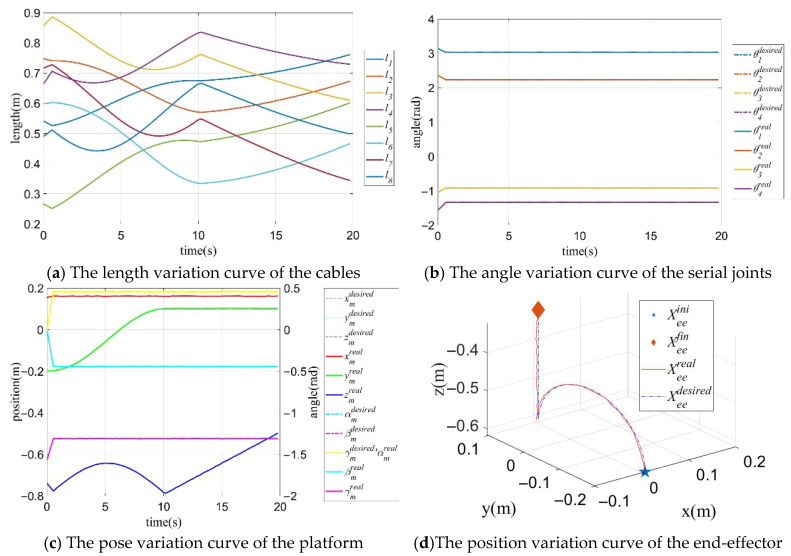
Trajectory variation curves of the mobile platform pose, generalized joints and the end-effector with Case C.

In the “2”-type trajectory tracking process, the pose variation curves of the mobile platform are shown in Figure 20a,b, the variation curves of the generalized joint are shown in Figure 20c, and the comparison results of the actual trajectory and the desired trajectory of the CDHR are shown in Figure 20d. Figure 20 shows that the poses of the mobile platform and arm shape of the RSR are motional complementary.

As summarized in Table 9, the maximum position 2-norm error is 0.044–0.048 mm, and the maximum attitude 2-norm error is 0.0147–0.0179° across the three representative trajectories. These errors are significantly smaller than typical tolerances required for platform positioning and end-effector manipulation in our prototype-scale tasks, indicating that the proposed method achieves stable and accurate tracking performance in simulation.

### 8.2. Experiment Study

#### 8.2.1. Experiment System Setup

**Figure 21:**
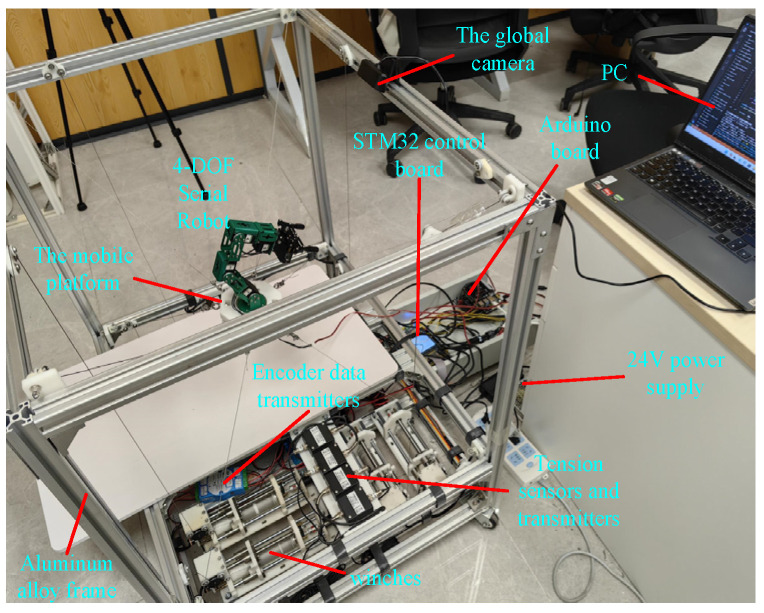
The CDHR prototype.

As shown in Figure 21, the CDHR prototype consists of a CDPR with eight cables, a moving platform, a 4-DOF SM, and two vision systems. Fixed housing and bottom hardware support beams were assembled using aluminum alloy materials from the Euro-standard 3030 series, along with the corresponding angle brackets and L-shaped aluminum alloy connectors. The tension sensor support plate was machined from high-strength wear-resistant 304 stainless steels. The motors used to drive the winches were MAXON RE25 models equipped with 500-line encoders. The motor reduction ratio was approximately 128, whereas the remaining motors exhibited a reduction ratio of approximately 372. Incremental encoders were connected to the transmitters with RS485 transceiver chips, and the lower controller read the encoder counts stored in the transmitters.

Owing to the differences in the cable winding angles and input impedance of the voltage acquisition channels, the linear ratio between the tension of each cable and the corresponding sensor’s output analog voltage varies. To control the cable tension more accurately, it was necessary to calibrate the parameters of the tension sensors prior to starting the experiment. The specific calibration experiment is detailed in Supplementary Information.

#### 8.2.2. Self-Calibration Experiment of System Parameters

**Figure 22:**
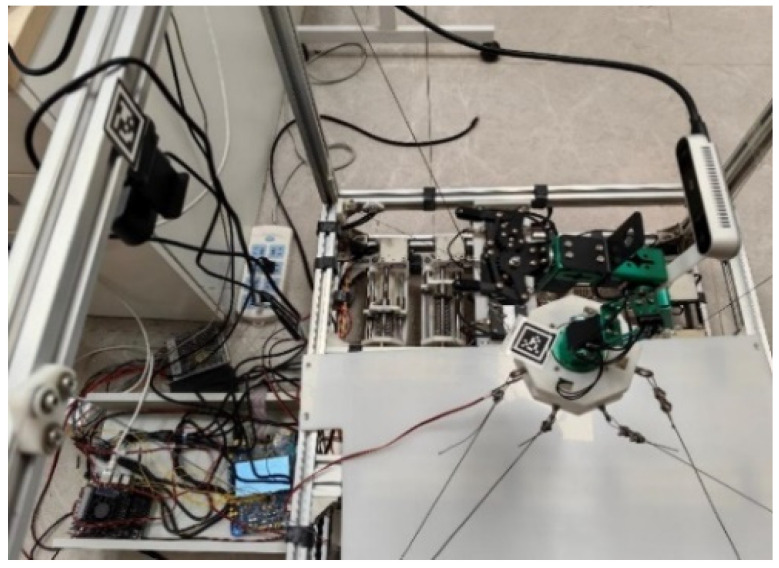
Calibration scene of the CDHR.

Before starting the experiment, the QR code for calibration was mounted on an aluminum frame, as shown in Figure 22. The number of calibration datasets was chosen as $N_{\mathrm{calib}}=15$, and the initial pose of the moving platform was set as $X_{m0}={\left[0.3350m,-0.0120m,-0.3550m,0,0,0\right]}^{T}$. Based on Equation (16), the initial cable lengths were calculated as $l_{0}={\left[0.5808,0.5905,0.5902,0.5815,0.5092,0.5307,0.5301,0.5093\right]}^{T}$. The initial joint angles of SM were $\Theta_{s0}=\left[0,2.2689,-1.0472,-1.0472\right]\left({}^{\circ}\right)$. The joint angle variation was within the range of [−15°, 15°]. Based on the modeling in Section 6, the self-calibration results of the system are shown in Table 10. The identified parameters in Table 10 provide the geometric mappings among the fixed anchor seats, the moving platform, and the hand–eye system, which are required for accurate trajectory planning and control. Although ground-truth values are not directly available in the physical experiment, the effectiveness of the self-calibration is reflected in the subsequent trajectory tracking results, where the end-effector paths closely follow the desired ones under the calibrated structural parameters (see Table 11).

#### 8.2.3. Trajectory Tracking Experiment

The CDHR was commanded to track three representative trajectories: a straight line (Case A), an arc (Case B), and a “2”-type trajectory (Case C). The “2”-type trajectory parameters are shown in Table 12.

**Figure 23:**
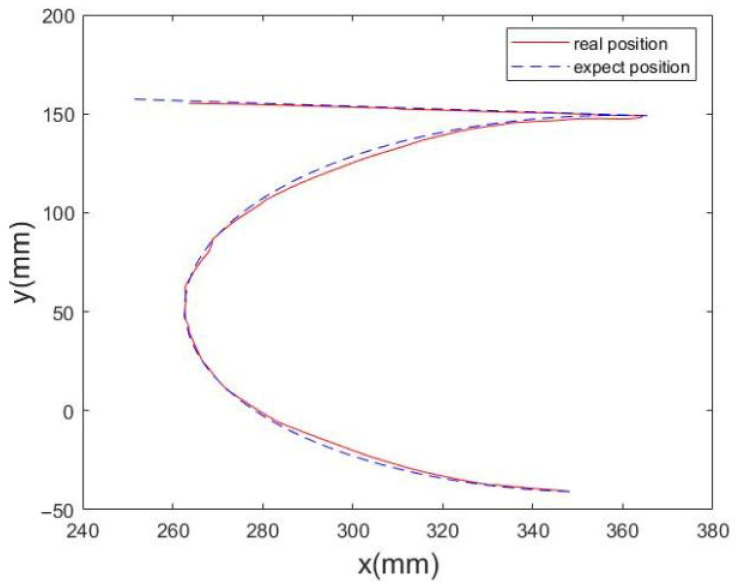
Comparison of actual end-effector pose vs. desired end-effector pose.

**Figure 24:**
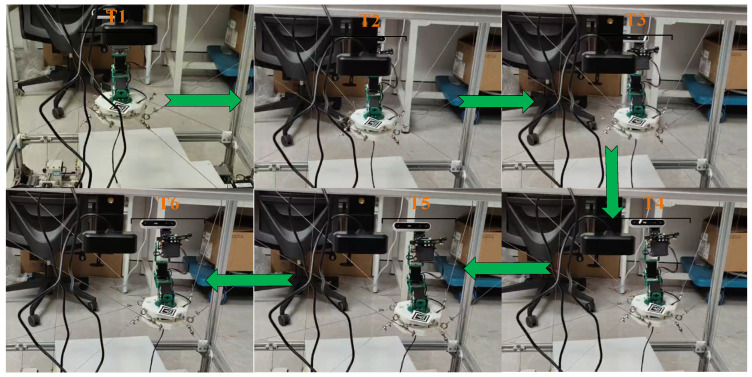
Process diagram of the trajectory tracking experiment.

**Figure 25:**
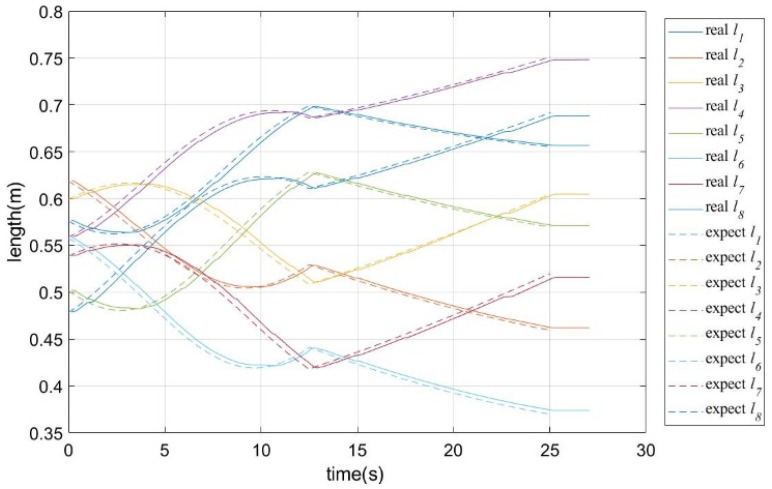
Comparison of desired cable length vs. actual cable length.

The results of the several typical trajectories are summarized in Table 11. As shown in Figure 23, during the motion process, the rate of change in the actual cable length closely matched the expected rate of change (i.e., the maximum cable length error was 2.1582 mm). The desired pose of the moving platform also closely matched the actual pose (i.e., the maximum position error was 3.2751 mm and the maximum orientation error was 1.2193°). The measured end-effector paths closely follow the desired ones, and the pose variation in the mobile platform remains small, indicating that the platform motion and the arm deformation are still kinematically complementary in real hardware. Based on the trajectory tracking method described in Section 7, the state diagram of the tracking process is shown in Figure 24, and the comparison between the desired and actual cable length curves is shown in Figure 25. The experimental results indicate that there is a lag between the desired and actual cable lengths, which is primarily caused by the communication delays between the upper and lower control systems. We found that the trajectory data on the upper computer is generated according to the upper-computer timeline, whereas the actual cable lengths on the lower computer are sampled according to the lower-computer timeline. A time offset exists between the two, and communication between the upper and lower computers incurs latency, which causes the measured cable lengths to lag behind the desired cable lengths. Based on the literature on distributed robotic control and embedded communication [36,37], the observed lag between desired and measured cable lengths is consistent with typical PC–microcontroller systems communicating over serial/fieldbus links without hardware-level clock synchronization. For example, experiments on Ethernet-based and CAN/serial robot controllers report end-to-end communication delays on the order of 5–20 ms, and show that a 10 ms delay can shift the measured joint/cable states by about 1–2 sampling periods when the control loop runs at 100–200 Hz, producing an apparent phase lag in position or length responses. These works further confirm that unsynchronized clocks between master and slave controllers lead to timestamp misalignment and additional effective delay in logged data, which directly manifests as the “delayed” actual trajectory relative to the reference one. It should be noted that the current implementation assumes reliable sensor feedback (e.g., encoder readings and continuous marker visibility for vision-based measurements) and uses the adopted modeling assumptions in Section 4 and Section 7. For systems with higher DOFs (more cables/joints) or heavier payloads, the calibration and optimization variables will increase, potentially raising computational cost and sensitivity to unmodeled effects (e.g., cable elasticity and structural compliance). These aspects will be further addressed in future work by incorporating a full dynamic model and deformation compensation.

## 9. Discussion

The CDHR shows promising potential in fields such as rapid spatial transportation and autonomous control in complex environments. In this study, we propose a CDHR prototype with high integration and a compact structure. The moving platform and the on-platform SM are optimized for operation in confined and complex spaces. A kinematic modeling method for a hybrid robot was proposed, incorporating both serial and parallel elements, and a corresponding static model was developed. Additionally, the force-coupling effects between the SM and moving platform under collision constraints, along with the force-closure conditions of the hybrid robot, were analyzed. The workspace of the hybrid robot is examined under various conditions. Based on global vision and hand-eye coordination, a synchronous self-calibration method was proposed for the multi-mapping relationships between fixed anchor seats, the arm platform, and hand-eye systems, addressing the time-consuming issue of step-by-step structural parameter calibration. Using the calibrated structural parameters, a trajectory tracking control method for the CDHR is presented. Finally, the performances of the prototype and proposed methods were fully validated using both the simulation system and physical prototype. Future work will build upon a full dynamic model of the CDHR to enable compliant hybrid force–motion control, which jointly regulates end-effector motion and contact force during physical interaction. We will adopt task-space impedance/admittance control to shape end-effector compliance: impedance control defines a desired dynamic relation between motion error and interaction force (virtual mass–spring–damper behavior), while admittance control maps measured force into motion commands. Compliance will be allocated across the hybrid system, with the CDPR responsible for low-frequency large-scale motions and the on-platform serial mechanism providing high-bandwidth fine dexterity. In parallel, we will model material flexibility by representing cable stretch, creep, and hysteresis together with platform and idler compliance, and we will compensate deformation through real-time stretch estimation from tension and length residuals.

## Figures and Tables

**Table 1:** Comparison of Modeling, Calibration, and Control Methods.

Methods	Modeling	Working Space Analysis	Calibration	Control
Harms et al. [30]	Hybrid climbing CDPR; Kinematic & static models; Non-modular.	-	-	-
Michelin et al. [31]	6-DOF CDPR + 7-DOF arm; Redundancy used for obstacle avoidance; Not modular.	-	-	Redundancy-based path following; Coupling-aware tension regulation not considered.
Song et al. [32]	Multi-pattern hybrid CDPR; Geometric and dynamic models; Parallel–serial force coupling neglected.	Geometric workspace analysis for two modes; No movable-anchor optimization.	-	-
An et al. [9]	CDPR with movable anchors (M-CDPR); Kinematics	Movable anchors; ≈456% increase in feasible workspace	-	Obstacle-aware motion planning; Parallel-only, no hybrid coupling control
Borgström et al. [25]	NIMS3D CDPR; Full kinematic	-	In-field self-calibration for Rapid deployment	Feedback tracking with optimized tension distribution

**Table 2:** Key symbols and variables.

Variable	Meaning
$\left\{G\right\}$, $\left\{M\right\}$, $\left\{S_{j}\right\}$, $\left\{B\right\}$, $\left\{E\right\}$	The global frame of the CDHR, the mobile platform frame, the *j*th joint frame of the SM, the base frame of the SM mounted on the moving platform, the tips frame for the CDHR
$O_{g}$, $O_{m}$, $O_{b}$, $O_{\mathrm{sj}}$, $O_{e}$	The origin of the CDHR global frame, the origin of the moving platform frame, the origin of the base frame for the SM mounted on the moving platform, the origin of the *j*th joint frame for the SM, the origin of the tips frame for the CDHR
$D_{c}$	Number of cables connected to the moving platform
$D_{s}$	Number of joints of the SM mounted on the moving platform
$D_{m}$	The DOFs of the moving platform
$D_{h}$	Generalized DOFs of the CDHR
$D_{e}$	The DOFs of the end-effector for the CDHR
$i=1∼D_{c}$	Index related to the number of cables
$j=1∼D_{s}$	Index related to the SM
$A_{i}$	Anchor seat connecting the moving platform to the *i*th cable (dynamic anchor seat)
$B_{i}$	Anchor seat connecting the frame to the *i*th cable (fixed anchor seat)
$p_{bi}$	Position vector of the fixed anchor seat relative to the global frame
$r_{ai}$	Position vector of the dynamic anchor seat relative to the mobile frame
$l_{i}$	Length of the *i*th cable
$L_{i}$	The direction vector of the *i*th cable, from the dynamic anchor seat to the fixed anchor seat
${\hat{L}}_{i}$	The unit direction vector of the *i*th cable
$l={\left[l_{1},l_{2},\ldots ,l_{D_{c}}\right]}^{T}$	The vector containing the lengths of all cables
$L={\left[L_{1},L_{2},\ldots ,L_{D_{c}}\right]}^{T}$	The matrix containing the direction vectors of all cables
$t_{ci}$	Tension magnitude of the *i*th cable
$T_{ci}$	Tension vector of the *i*th cable
$M_{ci}$	Moment caused by the tension of the *i*th cable
$p_{m}$	Position vector of the origin Om of the moving frame relative to the global frame {G}
$\theta_{j}$	The joint angle of the *j*th joint of the SM
$\Theta_{s}={\left[\theta_{1},\theta_{2},\ldots ,\theta_{D_{S}}\right]}^{T}$	The vector containing the joint angles of all joints in the SM
$\Theta_{w}={\left[\theta_{w1},\theta_{w2},\ldots ,\theta_{wD_{c}}\right]}^{T}$	The vector containing the rotation angles of all motors in the CDPR
$X_{m}={\left[x_{m},y_{m},z_{m},\alpha_{m},\beta_{m},\gamma_{m}\right]}^{T}$	Pose of the moving platform frame relative to the global frame

**Table 3:** Range of joint angles for the SM.

Name	θ (°)	*d* (m)	*a* (m)	α (°)
Joint 1	0	0.0500	1.5708	0
Joint 2	90	0	0.0829	0
Joint 3	0	0	0	0
Joint 4	0	0	0.1658	0

**Table 4:** Uniformly discretized sampled pose points.

Name	*x*-Axis	*y*-Axis	*z*-Axis
Minimum	0.06	−0.36	−0.62
Maximum	0.62	0.36	0
Spacing	0.02	0.02	0.02

**Table 5:** PSO parameters for optimizing the anchor seat distribution.

Maximum Iterations	Population Size	Personal Learning Factor	Global Learning Factor	Initial Inertia Weight	Final Inertia Weight
2000	100	2	2	0.9	0.4

**Table 6:** Optimized dynamic anchor seat position distribution.

Name	$A_{1}$ (m)	$A_{2}$ (m)	$A_{3}$ (m)	$A_{4}$ (m)	$A_{5}$ (m)	$A_{6}$ (m)	$A_{7}$ (m)	$A_{8}$ (m)
Position	$\left[\begin{array}{l} -0.0623\\ -0.0258\\ -0.0150 \end{array}\right]$	$\left[\begin{array}{l} -0.0258\\ 0.0623\\ -0.0150 \end{array}\right]$	$\left[\begin{array}{l} 0.0258\\ 0.0623\\ -0.0150 \end{array}\right]$	$\left[\begin{array}{l} 0.0623\\ -0.0258\\ -0.0150 \end{array}\right]$	$\left[\begin{array}{l} -0.0258\\ -0.0623\\ 0.0150 \end{array}\right]$	$\left[\begin{array}{l} -0.0623\\ 0.0258\\ 0.0150 \end{array}\right]$	$\left[\begin{array}{l} 0.0623\\ \begin{array}{l} 0.0258\\ 0.0150 \end{array} \end{array}\right]$	$\left[\begin{array}{l} 0.0258\\ \begin{array}{l} -0.0623\\ 0.0150 \end{array} \end{array}\right]$

**Table 7:** Simulation results of self-calibration.

Variable	True Value		Measured Average Value		Error ΔX	
	Position (mm)	Orientation (°)	Position (mm)	Orientation (°)	Position (mm)	Orientation (°)
pb1g	−334.00 −370.00 −900.00	-	−334.05 −370.03 −900.04	-	0.07	-
pb2g	−334.00 385.00 −885.00	-	−334.03 385.01 −885.04	-	0.05	-
pb3g	−334.00 370.00 −280.00	-	−334.03 370.02 −280.09	-	0.10	-
pb4g	−334.00 −385.00 −295.00	-	−334.04 −385.02 −295.08	-	0.09	-
pb5g	316.00 −370.00 −900.00	-	316.04 −370.07 −900.02	-	0.08	-
pb6g	316.00 385.00 −885.00	-	316.03 385.04 −855.02	-	0.03	-
pb7g	316.00 370.00 280.00	-	316.07 370.01 −280.06	-	0.05	-
pb8g	316.00 −385.06 −295.10	-	316.02 −385.10 −295.08	-	0.05	-
Xpb	0 0 0	−0.04 −0.04 −180.00	−0.03 −0.06 −0.07	−0.04 −0.06 −180.07	0.10	0.07
Xev	6.30 38.39 92.70	−89.22 −89.22 −90.79	6.29 38.40 92.61	−89.25 −89.25 −90.81	0.09	0.05

**Table 8:** Parameters of several typical trajectories.

Name		Trajectory Equations
**Case A**	Straight line trajectory	$\left\{\begin{array}{l} X_{\mathrm{ee}}^{\mathrm{ini}}={\left[0.0074,\;-0.1995,\;-0.6164,\;1.5732,\;-1.3077,\;-0.0025\right]}^{T}\\ X_{\mathrm{ee}}^{\mathrm{fin}}={[-0.2925785,\;0.1005394,\;-0.3163925,\;1.5732,\;-1.3077,\;-0.0025]}^{T} \end{array}\right.$ where $X_{\mathrm{ee}}^{\mathrm{ini}}$$\;\mathrm{and}\;X_{\mathrm{ee}}^{\mathrm{fin}}$ are the starting pose and the ending pose of the end-effector, respectively.
**Case B**	Spiral trajectory	$\left\{\begin{array}{l} X_{\mathrm{ee}}^{\mathrm{ini}}={\left[0.0074,\;-0.1995,\;-0.6164,\;1.5732,\;-1.3077,\;-0.0025\right]}^{T}\\ X_{\mathrm{center}}={\left[-1.9926,\;0,\;-0.5000,\;1.5732,\;-1.3077,\;-0.0025\right]}^{T}\\ x_{\mathrm{ee}}=0.0074-t_{i}\cdot h_{\mathrm{step}}\\ y_{\mathrm{ee}}=r_{\mathrm{ctraj}}\cdot \cos\left(\theta_{\mathrm{ini}}+t_{i}\cdot \theta_{\mathrm{step}}\right)\\ z_{\mathrm{ee}}\;=\;-0.6164-r_{\mathrm{ctraj}}\cdot \sin\left(\theta_{\mathrm{ini}}+t_{i}\cdot \theta_{\mathrm{step}}\right)\\ \alpha_{\mathrm{ee}}=1.5732\\ \beta_{\mathrm{ee}}=-1.3077\\ \gamma_{\mathrm{ee}}=-0.0025 \end{array}\right.$ where $X_{\mathrm{ee}}^{\mathrm{ini}}$$\;\mathrm{and}\;X_{\mathrm{ee}}^{\mathrm{fin}}$ are the starting pose and the ending pose of the end−effector, respectively, $r_{\mathrm{ctraj}}$ is the radius of the circle, $r_{\mathrm{ctraj}}=0.2309$$,\;h_{\mathrm{step}}=0.01$$,\;\theta_{\mathrm{ini}}$ is the initial angle of the circle trajectory corresponding to $X_{\mathrm{ee}}^{\mathrm{ini}}$$,\;\theta_{\mathrm{step}}=\pi /10$.
**Case C**	“2”-type trajectory	$\left\{\begin{array}{l} X_{\mathrm{ee}}^{\mathrm{ini}}={\left[0.0074,\;-0.1995,\;-0.6164,\;1.5732,\;-1.3077,\;-0.0025\right]}^{T}\\ X_{\mathrm{ee}}^{\mathrm{fin}}={\left[0.0074,\;0.1005,\;-0.3164,\;1.5732,\;-1,\;3077,\;\;-0.0025\right]}^{T} \end{array}\right.$ where the circular arc segment of shape 2 is an 1800 semicircle, and the remaining part is a straight-line segment.

**Table 9:** Simulation results of the several typical trajectories.

Categories	Name	Maximum Dimension Errors						Maximum 2-Norm Error	
		x (mm)	y (mm)	z (mm)	α (°)	β (°)	γ (°)	Position (mm)	Attitude (°)
Case A	Straight line trajectory	0.0460	0.0360	0.0190	0.0099	0.0062	0.0116	0.0480	0.0147
Case B	Spiral trajectory	0.0390	0.0300	0.0180	0.0124	0.0050	0.0129	0.0440	0.0179
Case C	“2”-type trajectory	0.0400	0.0300	0.0140	0.0117	0.0049	0.0128	0.0440	0.0172

**Table 10:** Self-calibration experimental results.

Value	Calibrated Value	
	Position (m)	Orientation (°)
pb1g	[0.3513, −0.0093, −0.6181]^T^	-
pb2g	[−0.3698, −0.0079, −0.5995]^T^	-
pb3g	[−0.3633, −0.0169, −0.0153]^T^	-
pb4g	[0.3817, −0.0198, −0.0288]^T^	-
pb5g	[0.3495, 0.6103, −0.6187]^T^	-
pb6g	[−0.3784, 0.6182, −0.6076]^T^	-
pb7g	[−0.3442, 0.6081, −0.0285]^T^	-
pb8g	[0.3575, 0.6032, −0.0533]^T^	-
Xev	[0.0235, 0.0198, 0.1066]^T^	[0.0344, −90.7450, 0.0344]^T^
Xmb	[−0.0190, −0.0061, −0.0093]^T^	[0.0688, 0.1375, 180.5963]^T^

**Table 11:** Results of the several typical trajectories.

Categories	Name	Maximum Dimension Errors						Maximum 2-Norm Error	
		x (mm)	y (mm)	z (mm)	α (°)	β (°)	γ (°)	Position (mm)	Attitude (°)
Case A	Straight line trajectory	0.1167	0.0091	0.0023	0.0169	0.3076	0.4794	0.1171	0.5689
Case B	Arc trajectory	0.1182	0.1899	0.0014	0.0154	0.3314	0.4338	0.1907	0.5462
Case C	“2”-type trajectory	0.1003	0.1958	0.0020	0.0181	0.3257	0.4902	0.2224	0.5887

**Table 12:** Starting point pose of the tracking trajectory.

Semicircular Arc Starting Point		Semicircular Arc Endpoint (Line Starting Point)		Line Segment Endpoint	
Position (m)	Attitude (°)	Position (m)	Attitude (°)	Position (m)	Attitude (°)
${\left[\begin{array}{l} 0.355\\ -0.040\\ -0.359 \end{array}\right]}^{T}$	${\left[\begin{array}{l} 0\\ 0\\ 0 \end{array}\right]}^{T}$	${\left[\begin{array}{l} 0.355\\ 0.150\\ -0.359 \end{array}\right]}^{T}$	${\left[\begin{array}{l} 0\\ 0\\ 0 \end{array}\right]}^{T}$	${\left[\begin{array}{l} 0.250\;\\ 0.150\\ -0.359 \end{array}\right]}^{T}$	${\left[\begin{array}{l} 0\\ 0\\ 0 \end{array}\right]}^{T}$

## Data Availability

Data are contained within the article. Further inquiries can be directed to the corresponding author.

## Supplementary Materials

The following supporting information can be downloaded at: https://www.mdpi.com/article/10.3390/s26041147/s1, Video S1: Simulation and Experimental Video of a Cable-Driven Hybrid Robot.
